# Regioselective synthesis of novel spiro-isoxazolines congeners as antimicrobial agents: *in vitro* and *in-silico* assessments

**DOI:** 10.3389/fchem.2025.1740409

**Published:** 2025-12-29

**Authors:** Rachid Bouzammit, Soumia Ait Assou, Mohammed Er-Rajy, Noura Aflak, Lahoucine Bahsis, Mohammed Chalkha, Mohammed El Hassouni, Mohammed Lachkar, Taibi Ben Hadda, Daryn Benson, Abdullah A. Alyousef, Mourad A. M. Aboul-Soud, John P. Giesy, Ghali Al Houari

**Affiliations:** 1 Engineering Laboratory of Organometallic, Molecular Materials and Environment (LIMOME), Faculty of Sciences, University Sidi Mohamed Ben Abdellah, Fez, Morocco; 2 Biotechnology, Environment, Agri-Food and Health Laboratory, Faculty of Sciences Dhar El Mahraz, Sidi Mohamed Ben Abdellah University, Fez, Morocco; 3 LIMAS Laboratory, Chemistry Department, Faculty of Sciences Dhar El Mahraz, Sidi Mohamed Ben Abdellah University, Fez, Morocco; 4 Team of Organic Chemistry and Valorization of Natural Substances, Faculty of Sciences, University Ibn Zohr, Agadir, Morocco; 5 Laboratory of Analytical and Molecular Chemistry/LCAM, Polydisciplinary Faculty of Safi, University Cadi Ayyad, Safi, Morocco; 6 Laboratory of Materials Engineering for the Environment and Natural Resources, Faculty of Sciences and Techniques, University of Moulay Ismail, Errachidia, Morocco; 7 Euro-Medeterranean University of Fes (UEMF), Fez, Morocco; 8 Clinical Laboratory Sciences Department, College of Applied Medical Sciences, King Saud University, Riyadh, Saudi Arabia; 9 Center of Excellence in Biotechnology Research (CEBR), College of Applied Medical Sciences, King Saud University, Riyadh, Saudi Arabia; 10 Department of Veterinary Biomedical Sciences and Toxicology Centre, Western College of Veterinary Medicine, University of Saskatchewan, Saskatoon, SK, Canada; 11 Department of Integrative Biology and Center for Integrative Toxicology, Michigan State University, East Lansing, MI, United States; 12 Department of Environmental Sciences, Baylor University, Waco, TX, United States

**Keywords:** 1,3-dipolar cycloaddition, regioselectivity, spiroisoxazoline, antimicrobial activity, *in silico* studies, reactions with azo Schiff bases, Petra/Osiris/Molinspiration analyses

## Abstract

**Introduction:**

A new class of spiroisoxazolines was efficiently synthesized through a regioselective cycloaddition between arylidene tetralone **1** and arylnitrile oxides **2**, characterized and assessed for their in vitro antimicrobial activity.

**Methods:**

The structures and regioselectivity of the obtained cycloadducts were confirmed by 1H, 13C-NMR, IR, elemental analysis, and mass spectrometry, and further supported by theoretical calculations that explained the reaction process and the regioselective results. The antimicrobial profile of the synthetized spiro derivatives was evaluated against the yeast Candida albicans, the Gram-postive bacteria (*Staphylococcus aureus and Bacillus subtilis*), and the Gram-negative bacteria (*Escherichia coli and Pectobacterium basiliensis*). In addition, in silico studies were carried out to rationalize the experimental findings and provide mechanistic insight.

**Results and Discussion:**

Two spiroisoxazolines, defined as **3b** and **c**, showed notable antimicrobial activity, producing inhibition zones between 8.33 ± 0.57 and 14.00 ± 2.00 mm. Compound **3b** was active against all tested strains and demonstrated ampicillin-comparable MIC values of 10 μg/mL against *E. coli*, *P. brasiliensis*, and *B. subtilis*. It showed moderate to weak activity against S. aureus (90 μg/mL) and C. albicans (300 μg/mL). Compound **3c** displayed selective activity toward Gram-positive bacteria with MIC values of 50 and 500 μg/mL against *B. subtilis* and *S. aureus*, respectively. Molecular docking studies confirmed the high binding affinities of **3b** and **3c** toward the active sites of the targeted proteins, in agreement with the antimicrobial results. POM analyses further indicated the coexistence of antifungal (O1δ−—O2δ−) and antiviral (O1δ−—N1δ−) pharmacophoric sites, although steric constraints introduced by two methyl substituents may limit their optimal interaction. The calculations also confirmed favorable bioavailability and the absence of predicted toxicity for all compounds. Overall, this combined experimental -theoretical study highlights the mechanistic basis and biological relevance of these spiroisoxazolines, underscoring their potential as promising scaffolds for the rational design of antiviral drug candidates.

## 1 Introduction

Heterocycles play a fundamental role in organic chemistry, pharmaceuticals, biology, and materials science. They constitute the core framework of a wide range of compounds with significant chemical, biological, pharmacological, and industrial applications (Mezgebe and Mulugeta, 2022; Uppadhayay et al., 2022). Their structural versatility and tunable reactivity make them indispensable to modern chemistry. Representing nearly two-thirds of all known organic compounds, heterocycles are crucial in the discovery and development of bioactive molecules (Cao et al., 2017).

Due to their pharmacological (Shinde et al., 2024), medicinal (Wang et al., 2023), and industrial (Guo et al., 2019) potential, characterized by their exceptional reactivity and their ability to rapidly develop biologically active compounds, as a class of heterocycles, isooxazoline derivatives have attracted significant interest from chemists. This has led to a focus on the synthesis of new isooxazoline compounds to enhance their biological efficacy (Chalkha et al., 2022). Among the methods for the synthesis of isoxazoline compounds, 1,3-dipolar cycloaddition (1,3-DC) reactions are particularly noteworthy. Although other methods exist (Paciorek et al., 2022), these reactions represent the most versatile, widely used, and straightforward means of obtaining five-membered heterocyclic compounds containing oxygen and nitrogen atoms (Rana and Ansari, 2023).

**FIGURE 1:**
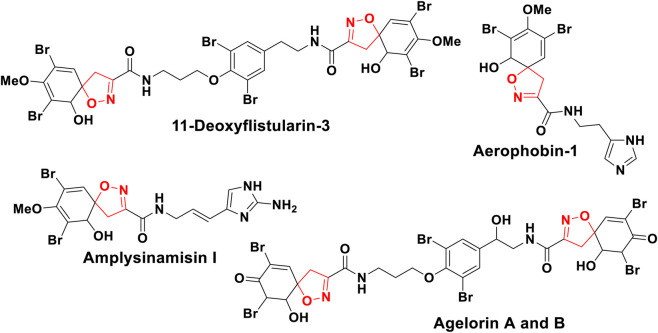
Biologically active natural products containing spiroisoxazoline structures.

The *spiro* function attached to a single carbon is a common structural motif found in many compounds with significant biological activity (Hong and Wang, 2013), such as anticancer (Aljohani et al., 2022), antitumor (Das et al., 2019; Najim et al., 2010), anti-tubercular (Mane et al., 2021), anti-inflammatory (Afsar et al., 2024), antifungal (Sawhney et al., 2022), and antiviral (Das et al., 2020). Due to their wide range of potential pharmacological applications, spiroisoxazolines are particularly important spiro-heterocyclic compounds in organic synthesis (Kumar et al., 2024). Some spiroisoxazoline compounds, such as 11-deoxyfistularin-3 (Ferreira Montenegro et al., 2024), and fluoro-substituted spiro-isooxazolines (Das et al., 2020), have demonstrated cytotoxic activity against cancer. Other Spiro compounds of this family, including aplysinamisin-1 (Rani et al., 2021), agelorin A, B (Moriou et al., 2021), derivatives of (R)–carvone (Dai et al., 2016), derivatives of (−)-α-santonin (Kaur et al., 2017), derivatives of artemisinin (Pratap et al., 2019) and Aerophobin-1 (Carnovali et al., 2022), have shown Antimicrobial (Raju et al., 2024), anticancer (Kalhor et al., 2024), antioxidants (AL-Adhreai et al., 2022) or antiproliferative properties (Alminderej et al., 2023) (Figure 1). However, despite these advances, previous studies still present several limitations. Many reported synthetic procedures suffer from limited regioselectivity, narrow substrate scope, or require harsh conditions. In addition, several spiroisoxazoline derivatives exhibit only moderate antimicrobial activity (Madadi Mahani et al., 2025), and very few studies have explored the incorporation of tetralone units into spiroisoxazoline frameworks. Moreover, mechanistic understanding remains incomplete, as only a limited number of publications combine experimental synthesis with computational approaches such as DFT, molecular docking, or POM theory. These gaps highlight the need for new spiroisoxazoline derivatives supported by a deeper mechanistic and biological analysis.

Due to the outstanding biological properties of isooxazoline units and spiro heterocyclic compounds (Raju et al., 2024), and consistent with our ongoing research efforts, focused on the synthesis of new heterocyclic systems intended for therapeutic use (Bouzammit et al., 2024a; Bouzammit et al., 2024b; Bouzammit et al., 2024c; Bouzammit et al. 2025; Ech-chihbi et al., 2025; Kanzouai et al., 2023; Wang et al., 2021), the main objective of the current study was to synthesize novel spiro heterocyclic derivatives containing isooxazoline and (Bouzammit et al., 2025) tetralone units. The resulting spiroisoxazolines were subsequently evaluated for their potential *in vitro* antibacterial activity against specific pathogenic microbial strains. In addition, *in silico* studies including ADME-T predictions, molecular docking simulations, and POM analysis were performed to support and explain the experimental results obtained.

## 2 Materials and methods

The Supplementary Material file provides a complete description of the general information, including solvents, reagents, and instruments, employed during the syntheses of spiroisoxazolines, and characterization of each compound.

### 2.1 Computational methods

All optimized molecular structures were calculated through the program “Gaussian 09” (Frisch et al., 2009) and use of the B3LYP/6-31G (d,p) basis set (Lee et al., 1988). Chloroform was used as the solvent in the polarizable continuum model (COCM). Indexes of chemical hardness (µ)/electronic chemical potential (η) were calculated (Equations 1, 2).

$$\eta =\left(E_{\text{LU}}-E_{\text{HO}}\right)$$ (1)

$$µ=\left(E_{\text{HO}}+E_{\text{LU}}\right)/2$$ (2)

Where LU is LUMO and HO is HOMO. The expressions of global nucleophilicity (*N*) and electrophilicity (ω) indexes were calculated (Equations 3, 4)

$$N=E_{\text{HO}}\;\left(\text{Nu}\right)-E_{\text{HO}}\;\left(\text{TCE}\right)$$ (3)

ω = µ^2^/2η (Chattaraj et al., 2006).

Local nucleophilic (P_k-_) and electrophilic (P_k+_) indices were found by the obtained values of the Mulliken atomic spin density of each reagent (Chattaraj et al., 2006). Consequently, the redefinition of the local nucleophilicity (*N*_k_) and electrophilicity (ω_k_) indices were defined (Equations 5, 6) (Domingo et al., 2013).

$$N_{k}=N\cdot P_{k}$$ (4)

$$\omega_{k}=\omega \cdot P_{k}^{+}$$ (5)

The ELF study was conducted using the Multiwfn software (Lu and Chen, 2012).

### 2.2 *In vitro* antimicrobial assay

#### 2.2.1 Agar-well diffusion method

The antimicrobial activity of spiroisoxazolines was evaluated against various microbial strains, including Gram^+^ bacteria (*Staphylococcus aureus* ATCC 29213 and *Bacillus subtilis* ATCC 6633), Gram^−^ bacteria (*Escherichia coli* K12 and *Pectobacterium brasiliensis* 13471), and the yeast *Candida albicans* ATCC 10231 through the agar-well diffusion technique. The working solution of each compound was prepared in dimethyl sulfoxide (DMSO, Sigma-Aldrich) at a concentration of 25 mg/mL. For the antimicrobial test, tested strains were grown in Muller Hinton (MH) broth for bacteria and in Sabouraud broth for *C*. *albicans.* Subsequently, a culture equivalent to 0.5 McFarland was employed. A total of 100 μL of this culture was combined with 5 mL of MH soft agar medium (0.5% (w/v) agar) for bacteria or with 5 mL of Sabouraud soft agar (0.5% (w/v) agar) for *C*. *albicans*. This microbial suspension was then spread evenly onto the surface of either MH agar or Sabouraud agar, based on the microorganism being tested (Hockett and Baltrus, 2017). Once the microbial overlay had set, a sterile tip was used to create a 6 mm diameter hole, into which 100 µL of the working solution, was added. The Petri dishes were incubated for 24 h, either at 37 °C for bacteria or at 30 °C for *C*. *albicans*. Wells that had ampicillin (2.5 mg) and amphotericine B (2.5 mg) served as positive controls for bacterial strains and *C*. *albicans*, respectively, while DMSO was used as the negative control. Inhibitory effect was assessed by measuring the inhibition zone diameter (IZD) around the growth. The experiment was conducted in triplicate, and the mean IZD value was determined.

#### 2.2.2 Minimum inhibitory concentration (MIC)

The MIC was evaluated by employing the microdilution method, adhering to the guidelines outlined in (Pfaller et al., 2002) and the protocol described in (Ait Assou et al., 2024). In a 96-well microplate, each well was filled with the appropriate culture medium, a suitable test concentration, and about 10^5^ cells of the tested bacteria or 10^3^ cells of *C. albicans.* Stock solutions were prepared for compounds 3 and 4 with concentrations of 25000, 10000, 9000, 8000, 7000, 6000, 5000, 4000, 3000, 2000, 1000, 900, 700, 500, 400, 300, 200, 100, 50, and 25 μg/mL. To achieve the required concentration of 2500, 1000, 900, 800, 700, 600, 500, 400, 300, 200, 100, 90, 70, 50, 40, 30, 20, 10, 5, and 2.5 μg/mL, 10 μL of the stock solution was added to each well, followed by the addition of the inoculum. Well 11, containing culture medium and inoculum, and well 12, containing only culture medium, served as the positive and negative controls for growth, respectively. The microplates were incubated at either 37 °C for bacteria or 30 °C for *C*. *albicans* for a duration of 24–48 h. After this incubation period, 20 μL of a 0.01% resazurin solution was introduced into each well, and the microplate was returned to the incubator at 30 °C for an additional 3 h to check the results. The growth of the microbial strains was indicated by a pink color, and the MIC was determined as the least concentration of the compound that did not result in pink color.

### 2.3 Molecular docking study

Molecular docking was used to determine the ligand-receptor interaction mechanisms involved in the complex (Er-rajy et al., 2025). Compounds **3b** and **3c** were synthesized and designed using ChemDraw 3D 16.0, and their geometry was optimized using the MM2 method (Mills, 2006). Discovery Studio 2021 software was then used to analyze the interactions between ligands and the protein receptor, removing water molecules, correcting missing side-chain residues and fusing non-polar hydrogens (Barghady et al., 2024; Systèmes, 2024). After preparing the protein and ligand, AutoDock Tools software was used to perform the molecular docking (Trott and Olson, 2010). Lamarck’s genetic algorithm was employed to perform the docking studies, aiming to obtain the lowest binding free energy (Er-Rajy et al., 2024). In AutoDockTools, preparation of the complex involved adding polar hydrogens and Kollman charges to the protein, generating Gasteiger charges for the ligand after automatic definition of the rotating bonds, and defining a grid that covered the entire protein surface before saving both structures in PDBQT format, in order to perform blind molecular docking (Er-rajy et al., 2023). A total of 30 solutions were calculated in each case, employing a population size of 300. Based on the results of the biological assays, the following receptors were selected. In the antibacterial study, the *S. aureus* receptor, obtained from the Protein Data Bank (PDB ID: 3VSL), with a resolution of 2.40 Å (Yoshida et al., 2012) was used. A grid was created using the following parameters: X = 10.09, Y = −48.570, and Z = 24.870 Å. In the study of the second antibacterial activity, we used the *B. subtilis* receptor, obtained from the Protein Data Bank (PDB ID: 1OF0), with a resolution of 2.45 Å (Martins et al., 2002). For protein-ligand docking studies, a grid was created using the following parameters: X = −3.173 Å, Y = 33.746 Å, and Z = 42.253 Å.

### 2.4 Petra/osiris/molinspiration (POM) theory

To determine the physico-chemical factors controlling the bioactivity of potential medications such as antibacterial, antifungal, antiviral and anticancer pharmacophore sites, a mixed computational Petra/Osiris/Molinspiration (POM) based model of which all three classes are available online and are free of charge, was used.

## 3 Results and discussion

### 3.1 Synthesis of the spiroisoxazolines

**SCHEME 1:**
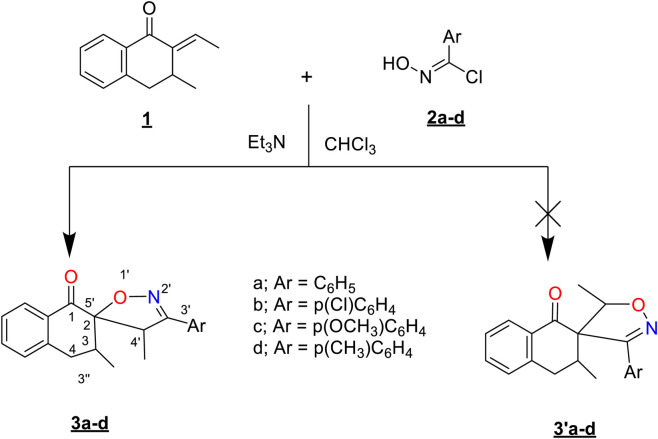
Spiro-isoxazoline synthesis from arylidene tetralone.

The dipolarophile **1** was prepared following the procedure described in our previously published work (Bouzammit et al., 2024c). Moreover, the dipoles **2** utilized in this study were synthesized by converting different aromatic aldehydes to their corresponding aldoximes (anti and syn), followed by a reaction with N-chlorosuccinimide in dimethylformamide (DMF) (Grundmann and Richter, 1968). Then, the obtained nitrile oxides **2** and (E)-2-ethylidene-3-methyl-3,4-dihydronaphthalene-1(2H)-one **1** were reacted in basic medium at room temperature, resulting in the diasterio- and regioselective formation of spirocycloadducts containing isoxazoline and tetralone units **3** (Scheme 1). The structures of the obtained cycloadducts are characterized by spectroscopic techniques and validated by mass spectrometry.

The analysis of FT-IR spectra of the synthesized spiroisoxazolines shows an absorption band between 1690 and 1700 cm^-1^, characteristic of the carbonyl (C=O) functional group. The assignment of the different signals in the ^1^H NMR spectra of the four compounds **3a-d** suggested the presence of two methyl groups (CH_3__**(3″)**_) and CH_3 (**4″)**_) appearing as successive doublets at 0.98 and 1.30 ppm, respectively. A multiplet at about 2.9 ppm corresponds to the proton H_3_, while the two chemically non-equivalent CH_2_ protons appear as two doublets of doublets located at about 2.7 and 3.7 ppm. Additionally, a quadruplet attributed to the proton H_**4’**_ of the isooxazoline ring appeared around 4.30 ppm, confirming the regiochemistry of compound **3.** These findings align well with the majority of the cycloadducts reported in the literature. In the case of the Regio-isomer **3′**, we would expect higher values above 6 ppm for the proton H_**5’**_ under the attractive effect of oxygen (Fihi et al., 1995). Compounds **3c** and **3d** show two distinct signals at 3.96 ppm and 2.41 ppm, respectively, indicative of the two methyl groups (Ar-OCH_3_) and (Ar-CH_3_). The regiochemistry of the resulted cycloadduct **3** is confirmed by interpreting data obtained from ^13^C NMR spectra. The attractive effect of oxygen is responsible for the chemical shifts observed for the spiranic carbon C_**2,5'**_, which are around 90 ppm (Akhazzanea et al., 2011). In contrast, the structure **3′**would predict much lower values for the spiranic carbon C_**2,4'**_, which is around 60 ppm (Fihi et al., 1995). The mass spectrometry data obtained are perfectly coherent with the proposed structures. The structure of the obtained products is validated using high-resolution mass spectrometry. All mass spectra of the synthesized spiroisoxazolines **3a-d** show a molecular ion peak [M + H]^+^ that corresponds exactly to the molecular mass of the proposed structure.

**SCHEME 2:**
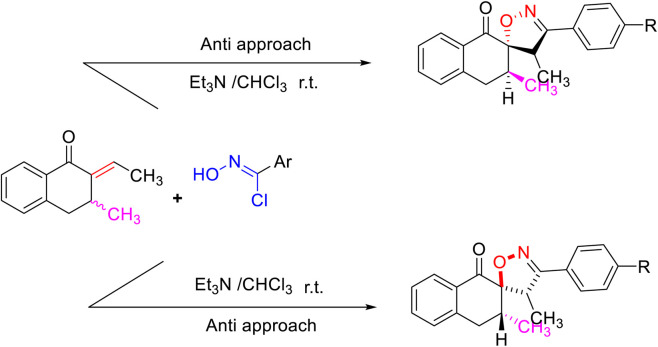
Regio- and diastereoselective formation of the spiroisoxazolines.

Overall, the 1,3-DC reaction of arylnitrile oxides with ethylenic dipolarophiles leads regioselectivity to 3,4-disubstituted isooxazolines **3** (Schemes 1 and 2) (Tóth et al., 1999). Additionally, the reaction proceeded in a diastereoselective manner, with the anti-approach being favored due to steric hindrance caused by the substituent CH_3_ at position 3 of the arylidene (Scheme 2) (Tóth et al., 1999).

### 3.2 Mechanistic study

**SCHEME 3:**
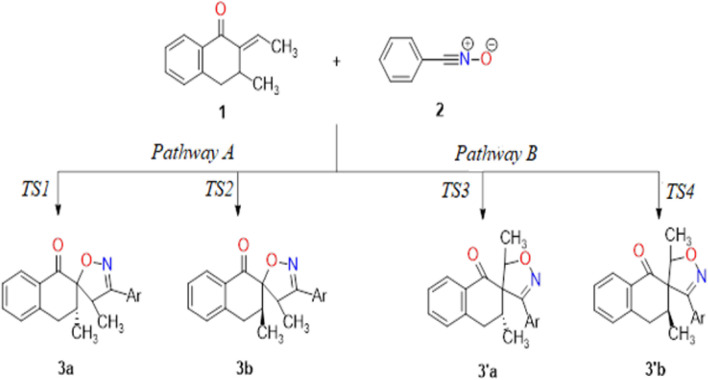
Plausible pathways for 1,3-DC reaction between nitrile oxide **2** and 2H-2-ethylidene-3-methyl-3,4-dihydronaphthalen-1-one **1**.

To explain the regioselectivity observed experimentally in this 1,3-DC reaction, ELF topological and MEDT analyses were carried out for theoretical studies. The 1,3-DC reaction, also known as 32CA, has gained acknowledgment as a remarkably efficient approach for producing a wide range of organic compounds with various practical uses (Ukaji and Soeta, 2014). Recent theoretical studies have confirmed the efficiency of this cycloaddition reaction, correlating it with the electronic structures of the three-atom components (TACs) involved in the [3 + 2] cycloaddition process (Ríos-Gutiérrez et al., 2021). In this work, the 1,3-DC reaction between 2*H*-2-ethylidene-3-methyl-3,4-dihydronaphthalen-1-one **1** and nitrile oxide **2** leads to the construction of the corresponding isooxazoline compound by two plausible paths concerning the regioselective attacks (Scheme 3) (Tu et al., 2022).

Recently, a theoretical investigation known as Molecular Electron Density Theory (MEDT) (Domingo, 2016) has been proposed to establish a robust link between three-atom components (TACs) and their interactions with ethylene compounds in 1,3-DC reactions (Ríos-Gutiérrez and Domingo, 2019b). To explain the observed regioselectivity in nitrile oxide and 2H-2-ethylidene-3-methyl-3,4-dihydronaphthalen-1-one, the MEDT approach was utilized, and the results were presented in four main sections. First, the reagents were analyzed using ELF topological analysis. Second, reactivity indices were examined using Conceptual Density Functional Theory (CDFT). Third, potential reaction profiles for the 1,3-DC reaction were investigated. Finally, ELF topological analysis was conducted on both reagents to reveal their ionic character.

#### 3.2.1 ELF study of reagents

**FIGURE 2:**
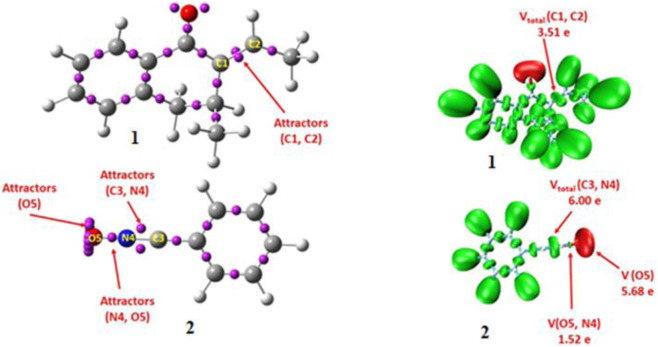
Basin attractors and corresponding isosurfaces for both reagents of ELF analysis.

The electronic nature of starting materials has been reported to significantly affect reaction pathways and the energetic barriers (Bahsis et al., 2020). Here, ELF functions were performed to analyze the electron density distribution and elucidate the chemical structures of reagents (Figure 2) (Becke and Edgecombe, 1990). To investigate the electronic features of the cycloaddition reaction between reagents **1** and **2**, an ELF analysis was performed on their optimized geometries (Figure 2). The ELF analysis of the optimized structures revealed two disynaptic basins associated with the C1-C2 bond in reagent **1**. These basins account for an electron population of 3.51 e, consistent with a typical carbon–carbon double bond. In compound **2**, the ELF topology shows two disynaptic basins along the C_3_≡N_4_ bond, containing a total of 6.00 electrons-indicative of a triple bond character. Additionally, a disynaptic basin on the N_4_-O_5_ bond integrates 1.52 e, and three monosynaptic basins are observed on the oxygen atom (O_5_), summing to 5.68 e. These observations support the characterization of the C_3_≡N_4_ linkage as a triple bond, the N_4_-O_5_ connection as a single bond, and the presence of three lone pairs on O_5_ (Domingo and Ríos-Gutiérrez, 2017). Overall, the results confirm that nitrile oxide derivatives act as zwitterionic 1,3-dipolar species in polar cycloaddition reactions (Figure 2) (Ríos-Gutiérrez and Domingo, 2019a).

#### 3.2.2 CDFT indices analysis of reagents

To explore the chemo- and regioselectivity of the reactions, global reactivity descriptors were applied, focusing on both the reactive sites and the nature of the interaction. This analysis was carried out within the conceptual DFT, including global electron density transfer (GEDT) as a key parameter (Domingo et al., 2022). The calculated global indices for both reactants are summarized in Table 1. According to this analysis, the nitrile oxide derivative **2** exhibits a higher electronic chemical potential (µ = −3.85 eV) compared to the 2*H*-2-ethylidene-3-methyl-3,4-dihydronaphthalen-1-one compound **1**, which has a µ of −4.24 eV. This energy difference suggests that during the transition state, electron density is moved from the nitrile oxide **2** toward the dihydronaphthalenone **1**. The computed nucleophilicity and electrophilicity values (Table 1) reveal that compound **2** behaves as a moderate nucleophile (*N* = 2.41 eV) and a moderately strong electrophile (*ω* = 1.45 eV). In contrast, compound **1** displayed moderate nucleophilicity (*N* = 2.16 eV) and a higher electrophilicity (*ω* = 1.86 eV), based on established reactivity scales (Domingo et al., 2002). These results indicate that 2*H*-2-ethylidene-3-methyl-3,4-dihydronaphthalen-1-one **1** acts predominantly as an electrophilic species in the 1,3-DC reaction, while the nitrile oxide **2** functions as the nucleophilic counterpart, indicating a polar reaction profile.

**TABLE 1:** Property and reactivity indices on a global scale. All measurements are expressed in electron volts (eV).

​	E_HOMO_	E_LUMO_	η	µ	ω	*N*
Dipolarophile 1	−6.66	−1.82	−4.24	4.84	1.86	2.16
Dipole 2	−6.41	−1.29	−3.85	5.13	1.45	2.41

**FIGURE 3:**
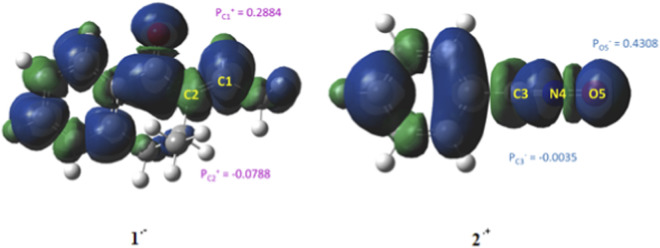
Isosurfaces *represent* Mulliken atomic spin densities with P_k_^+^ and P_k_^−^ for compound **1** and **2**, respectively. All reported values are given in electron volts (eV).

Recent analyses of local reactivity indices derived from Parr functions have revealed a strong correlation between bond formation in polar reactions and the experimentally observed chemo- and regioselectivity (Domingo, 2014). The study focused on examining the local nucleophilicity and electrophilicity (*N*_k_ and *ω*_k_, respectively) for nucleophilic and electrophilic reagents, respectively (Domingo et al., 2021). The computed values of the local electrophilicity for the 2*H*-2-ethylidene-3-methyl-3,4-dihydronaphthalen-1-one **1** and the local nucleophilicity for the nitrile oxide **2** are presented in Figure 3. The analysis indicates that carbon atom C_1_ in derivative **1** exhibits a slightly higher electrophilicity than carbon atom C_2_, while oxygen atom O_5_ in derivative **2** shows the highest nucleophilicity. These results indicate that the cycloaddition reaction between reagents **1** and compound **2** may occur via the interaction between the carbon atom C_1_ in reagent **1** and oxygen atom of reagent **2**.

#### 3.2.3 Reaction profiles for 1,3-DC reaction

**FIGURE 4:**
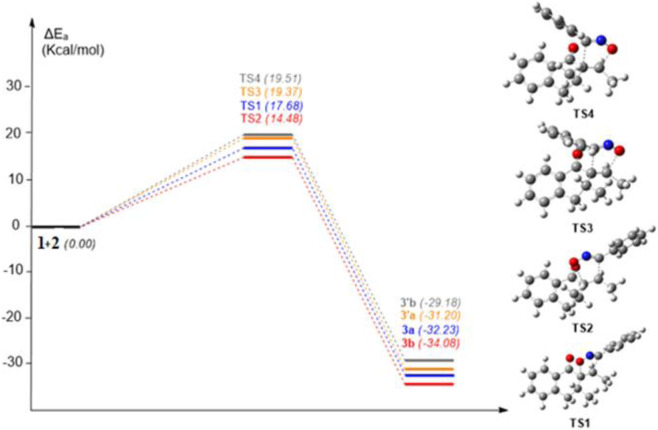
Activation energy diagram of *1,3-DC reaction* between reagents **1** and **2**, considering chloroform as the reaction medium. All energy values are reported in kcal/mol.

The next phase in this mechanistic investigation into the cycloaddition reaction between **1** and **2** aimed to explore the two possible reaction pathways via a single-step mechanism, resulting in the construction of four plausible products. Figure 4 summarizes the attributed activation energy values found through using chloroform as a solvent. Pathway A has a lower activation energy and greater stability than pathway B, even though the P_k_^+^ and P_k_^−^ Parr functions values suggest that pathway B is more favorable. These results may be attributed to the instability of **3’a**, and **3’b** products due to steric repulsions between the phenol groups (Figure 4). The results also suggest that TS2 has an activation energy of 14.48 kcal/mol, indicating slightly higher stability, with a difference of 3.2 kcal/mol compared to TS1. These transition states facilitate the formation of product **3a** in a more advantageous manner than **3b**.

### 3.3 Antimicrobial screening

#### 3.3.1 Well-diffusion assay

The antimicrobial activity of the synthesized spiro-isoxazoline derivatives was evaluated using the well diffusion method against Gram-positive and Gram-negative bacteria, as well as a yeast strain (Table 2). Among the tested compounds, **3b**, bearing a para-chloro substituent (p-ClC_6_H_4_), displayed the significant and broadest antimicrobial profile, with IZDs ranging from 8.33 ± 0.57 to 14.00 ± 2.00 mm. In comparison, the standard antibiotics ampicillin and amphotericin B produced significantly larger IZDs (26.66 ± 1.52 to 40.33 ± 0.57 mm). Compound **3c**, featuring a para-methoxy substituent (p-OCH_3_C_6_H_4_), showed selective antibacterial activity against *S. aureus* and *B. subtilis*, with IZDs ranging from 8.33 ± 1.15 to 9.33 ± 1.52 mm, while remaining inactive against the other microorganisms tested. These findings are consistent with established structure–activity trends, wherein electron-withdrawing groups such as chlorine enhance antibacterial potency by increasing lipophilicity and improving membrane permeation, whereas electron-donating substituents like methoxy typically confer reduced but sometimes more selective activity. Similar observations have been reported for chlorinated and methoxy-bearing isoxazolines and related heterocycles exhibiting antimicrobial properties (Raju et al., 2024).

**TABLE 2:** IZDs (mm) of the tested compounds.

Compounds	R	Antibacterial activity				Antifungal activity
		*E. coli*	*P. brasiliensis*	*S. aureus*	*B. subtilis*	*C. albicans*
**3a**	**p-C**_**6**_ **H**_**5**_	---	---	---	---	---
**3b**	**p-ClC**_**6**_ **H**_**4**_	14.00 ± 02.00^a^	12.33 ± 0.57	12.66 ± 00.57	14.00 ± 01.00	08.33 ± 00.57
**3c**	**p-OCH**_**3**_ **C**_**6**_ **H**_**4**_	---	---	08.33 ± 1.15	09.33 ± 01.52	---
**3d**	**p-CH**_**3**_ **C**_**6**_ **H**_**4**_	---	---	---	---	---
**Ampicillin**	**---**	23.00 ± 01.00	30.00 ± 01.00	27.66 ± 02.08	40.33 ± 00.57	---
**Amphotericin B**	**---**	---	---	---	---	26.66 ± 01.52

^a^ Values are expressed as mean ± SD.

#### 3.3.2 MIC assay

MIC values were determined for the two active compounds **3b** and **3c**. Compound **3b** exhibited remarkable inhibitory activity, with an MIC recorded at 10 μg/mL against *Escherichia coli,* comparable to that of ampicillin (10 μg/mL). Compound **3b** also showed a significant antibacterial effect against *Pectobacterium brasiliensis* and *B. subtilis* with MIC values of 10 μg/mL, compared to the positive control, with displayed MIC values of 5 μg/mL, and 2 μg/mL, respectively. Additionally, it showed moderate inhibitory action against *S. aureus (*90 μg/mL), and *Candida albicans (*300 μg/mL). Furthermore, **3c** displayed antibacterial effectiveness, with MIC values of 50 μg/mL against *B. subtilis* and 500 μg/mL against *S. aureus* (Table 3).

**TABLE 3:** MIC (µg/mL) for compounds **3b** and **3c**.

Compd	Antibacterial activity				Antifungal activity
	*E. coli*	*P. brasiliensis*	*S. aureus*	*B. subtilis*	*C. albicans*
**3b**	10	10	90	10	300
**3c**	---	---	500	50	---
**Ampicillin**	10	5	3	2	---
**Amphotericin B**	---	---	---	---	2

### 3.4 Molecular interaction analyses

**FIGURE 5:**
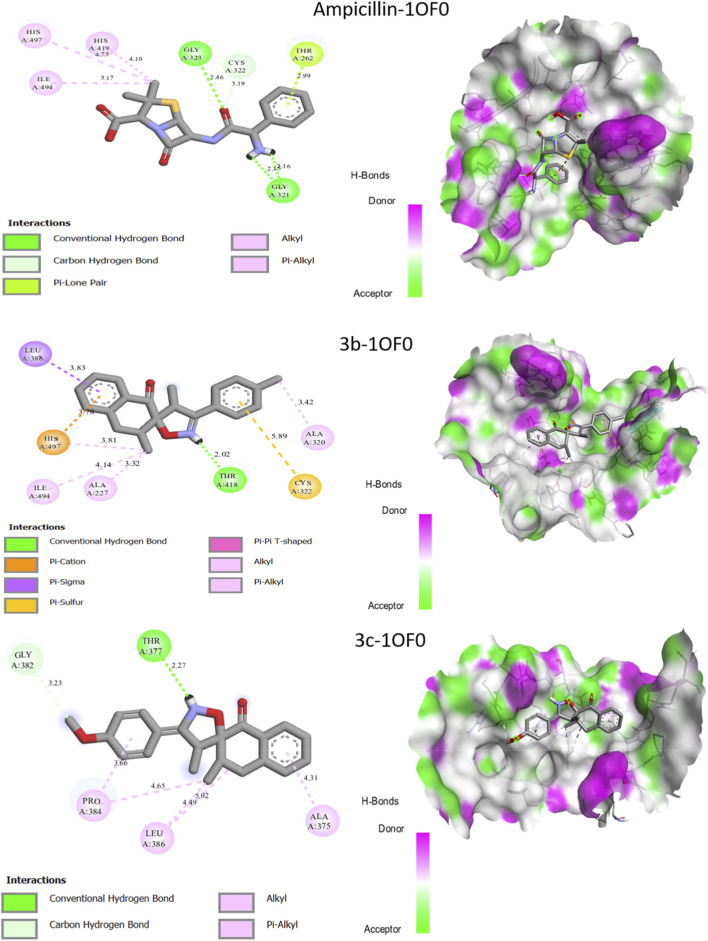
Interactions between the synthesized compound **3b** and **3c**, ampicillin and the 1OF0 receptor.

**FIGURE 6:**
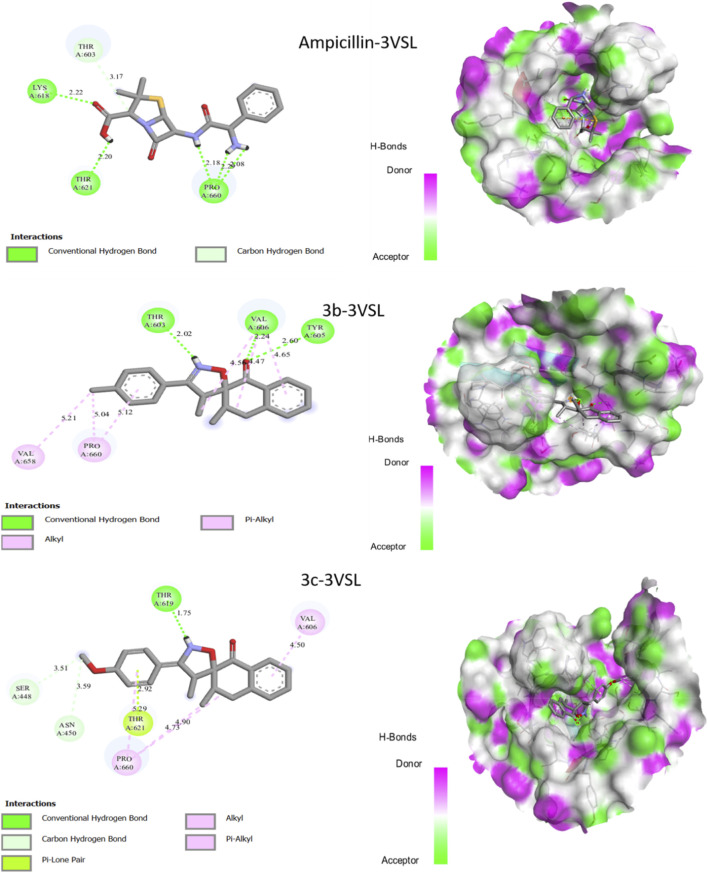
Interactions 2D between the synthesized compound **3b** and **3c**, ampicillin and the 3VSL receptor.

A molecular docking study for the synthesized compounds **3b** and **3c**, as well as for ampicillin was undertaken to explain its antibacterial activity against two Gram-positive strains, namely, *Staphylococcus aureus* and *Bacillus subtilis*. To better understand interactions between the two new molecules synthesized (**3b**, **3c**, and ampicillin) and the targeted proteins, molecular docking was performed to clarify how the chosen ligand interacts with its protein (Figures 5, 6; Table 4)**.**

**TABLE 4:** Molecular docking bending energy results for compounds **3b**, **3c**, and ampicillin against the two selected proteins.

Ligands	Complexes	Bending energy
**Ampicillin**	Ampicillin-1OF0	−7.80
**3b**	**3b**-1OF0	−8.23
**3c**	**3c**-1OF0	−6.62
**Ampicillin**	Ampicillin-3VSL	−7.37
**3b**	**3b**-3VSL	−9.33
**3c**	**3c**-3VSL	−8.19

The binding energy of the synthesized molecules and the drug ampicillin showed that the two new compounds (**3b** and **3c**) and the drug ampicillin have a good binding affinity (−6.62 and −9.33) with the two targeted proteins. Figure 5 shows the various molecular docking results with receptor 1OF0.

Molecular docking results for the **ampicillin-1OF0** complex (Figure 5) reveal two hydrogen bonds with residues Gly-223 and 321, at distances of 2.46 Å and 2.15 Å, respectively. In addition, a pi-lone pair interaction is present with residue Thr-226 at a distance of 2.99 Å. Three π-alkyl interactions are also present with residues His-419 and 497, and residue Ile-494, as well as a carbon-hydrogen bond with residue Cys-322.

Molecular docking results for the **3b-1OF0** complex (Figure 5) reveal a single hydrogen bond and single pi-cation with residues Thr-418 and His-497, at a distance of 2.02 Å and 3.78 Å respectively. In addition, a pi-sulfur interaction is observed with residue Cys-322, at a distance of 5.89 Å. Three π-alkyl interactions are also present with residues Ile-494, Ala-227 and 320. Similarly, molecular docking results for the **3c-1OF0** complex (Figure 5) reveal a single hydrogen bond with residue Thr-377, at a distance of 2.27 Å. In addition, a carbon hydrogen bond is observed with residue Gly-382, at a distance of 3.23 Å. Three π-alkyl interactions are also identified with residues Pro-384, Leu-386 and Ala-375. We observed that the two molecules synthesized, **3b** and **3c**, interact with the same residues as ampicillin, namely, threonine, cysteine and alanine. This confirms that these two compounds are indeed localized at the active site of the target protein. So, these interactions may contribute to the inhibition of the targets against *Bacillus subtilis*.

Molecular docking results for the three molecules studied show that all the selected molecules showed outstanding docking scores (Table 4) against antibacterial activity targeting *Staphylococcus aureus* (Figure 6).

Molecular docking results for the ampicillin-3VSL complex (Figure 6) reveal three hydrogen bonds with residues Pro-660, Thr-621, and Lyr-618, at distances of 2.08 Å, 2.20 Å, and 2.22 Å, respectively. Molecular docking results for the **3b-3VSL** complex (Figure 6) reveal three hydrogen bonds with residues Tyr-605, Val-606, and Thr-603, at distances of 2.60 Å, 2.24 Å, and 2.02 Å, respectively. In addition, two π-alkyl interactions are also present with residues Val-658 and Pro-660. Similarly, molecular docking results for the **3c**-**3VSL** complex (Figure 6) reveal two hydrogen bonds with residues Thr-619, and Thr-621, at distances of 1.75 Å, and 2.92 Å, respectively. In addition, a free π-electron interaction (π-lone pair) is observed with residue Thr-621, at a distance of 2.92 Å. Two π-alkyl interactions are also identified with residues Pro-606 and Val-606. We noted that the two molecules synthesized, **3b** and **3c**, interact with the same residues as ampicillin, namely, threonine and proline. This confirms that these two compounds are indeed localized at the active site of the target protein. Molecular docking studies against both bacterial strains revealed that the two synthesized compounds possess antibacterial potential and interact with amino acid residues like those targeted by the reference antibiotic, ampicillin.

### 3.5 POM analysis of compounds

**FIGURE 7:**
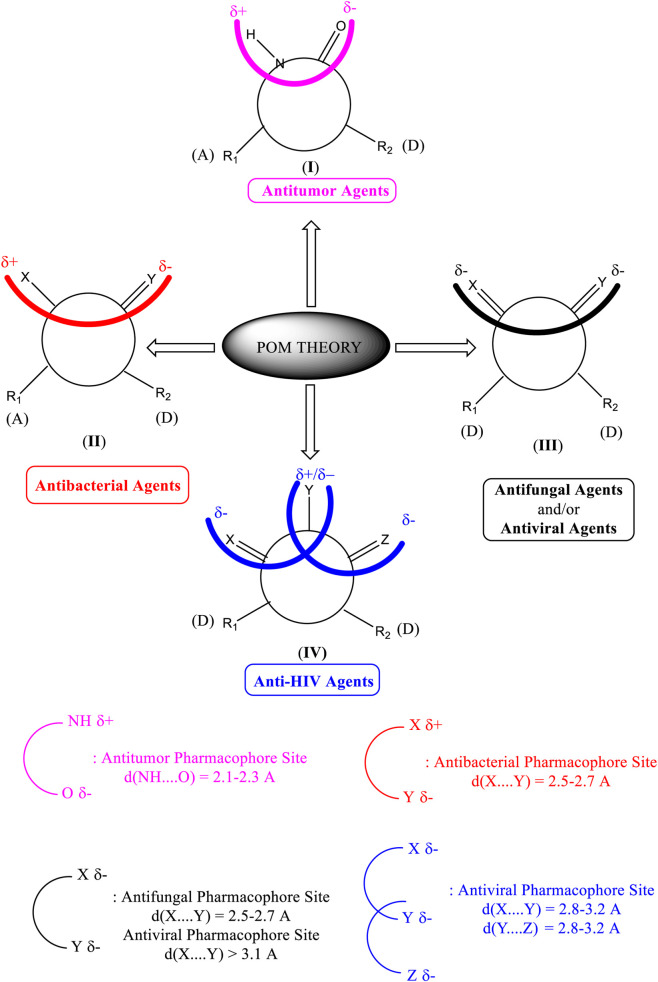
Organigram of POM Theory showing the geometry and atomic charge of pharmacophore site of antibacterial (Grib et al., 2020; Lakhrissi et al., 2022; Mabkhot et al., 2014; Rbaa et al., 2019), antifungal (Al-Maqtari et al., 2017; Rachedi et al., 2020), antiviral and antiparasitic (Aljofan et al., 2014; Jarrahpour et al., 2019; Kawsar et al., 2022; Khodair et al., 2021) and antitumor agents (Al-Maqtari et al., 2017; Aljofan et al., 2014; Bechlem et al., 2020; Bennani et al., 2013; Grib et al., 2020; Jarrahpour et al., 2019; Kawsar et al., 2022; Khodair et al., 2021; Lakhrissi et al., 2022; Mabkhot et al., 2014; Rachedi et al., 2020; Rbaa et al., 2019).

The POM Theory was developed by our group, in collaboration with NCI and TAACF of the United States of America. The principal goal is to demonstrate differences between various classes of commercial drugs, based on their physico-chemical properties and atomic charges of each pharmacophore site (Figure 7).

#### 3.5.1 Osiris calculations of toxicity and drug-score of compounds

When a structure is valid, the OSIRIS Property Explorer allows chemical structures to be determined and instantly calculates a variety of drug-relevant properties. The outcomes of predictions are colored-coded and rated. Red indicates properties that have a significant risk of undesirable outcomes, such as mutagenicity or poor intestinal absorption. Alternatively, drug-conformant behavior is indicated by a green hue (Table 5). None of the compounds of series **3a-d** have side effect and their drug score is encouraging (40%<DS<51%) but their bioavailability is not optimal (cLogP >5).

**TABLE 5:** Osiris calculations of toxicity and Drug-score of compounds **3a-d.**

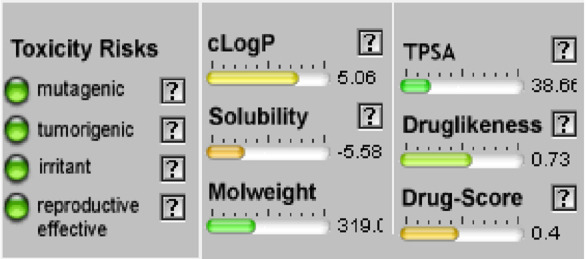	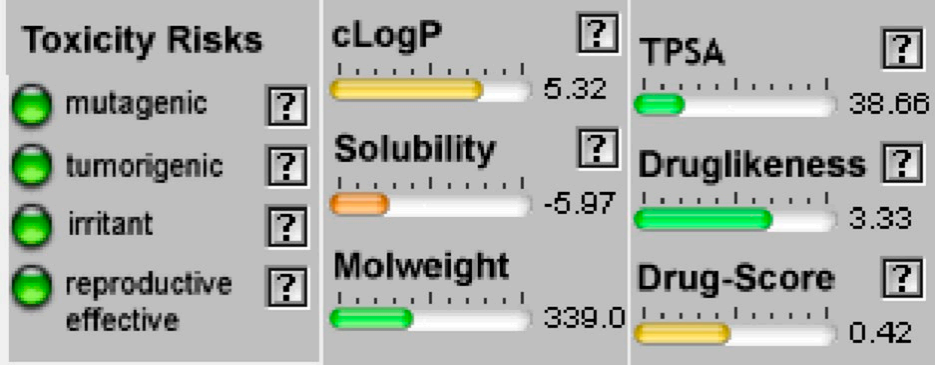
**Compound 3a**	**Compound 3b**
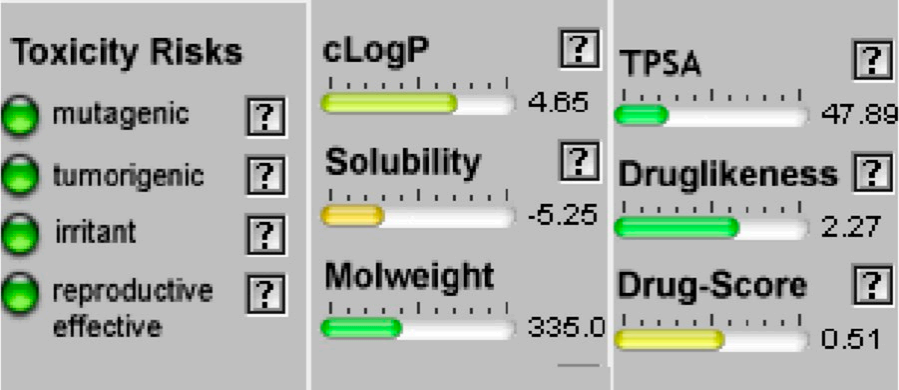	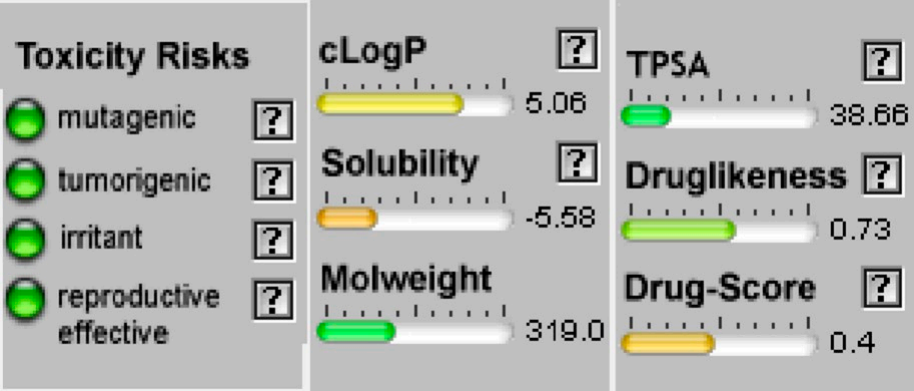
**Compound 3c**	**Compound 3d**

#### 3.5.2 Molinspiration calculations of molecular properties of compounds

To control of bioavailability of candidate drugs, it is of importance to calculate all parameters of Lipinski 5 rules, via Molinspiration program (Table 6). The consultation of Table 6 shows that all compounds meet the criteria of the bioavailability (NV < 2).

**TABLE 6:** Molinspiration calculations of physico-chemical properties of compounds **3a-d** according to Lipinski 5 rules.

Compd	3D structure		Lipinski 5 rules calculations
**3a**	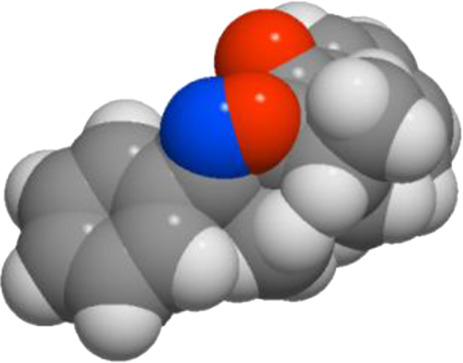	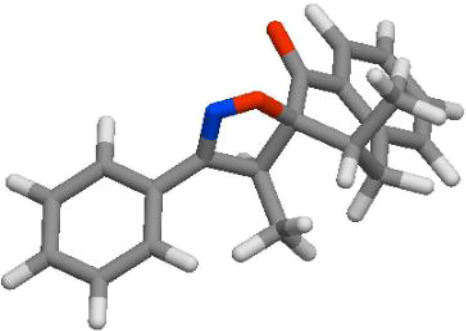	**miLogP 4.35** **TPSA 38.67** **natoms 23** **MW 305.38** **nON 3** **nOHNH 0** **nviolations 0** **nrotb 1** **volume 285.08**
**3b**	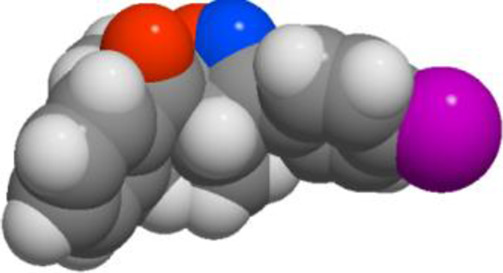	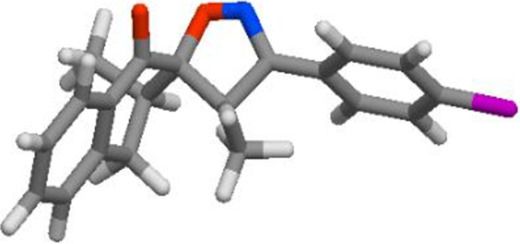	**miLogP 5.03** **TPSA 38.67** **natoms 24** **MW 339.82** **nON 3** **nOHNH 0** **nviolations 1** **nrotb 1** **volume 298.62**
**3c**	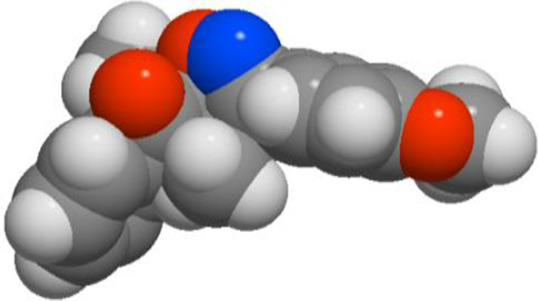	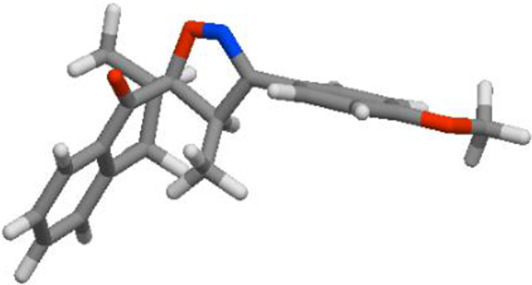	**miLogP 4.41** **TPSA 47.90** **natoms 25** **MW 335.40** **nON 4** **nOHNH 0** **nviolations 0** **nrotb 2** **volume 310.63**
**3d**	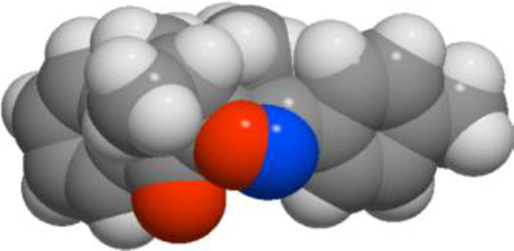	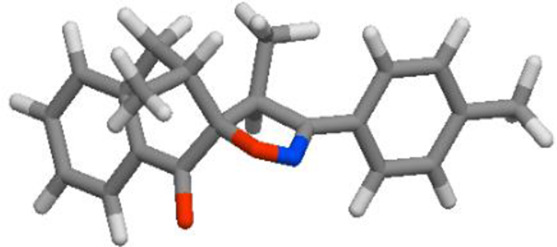	**miLogP 4.80** **TPSA 38.67** **natoms 24** **MW 319.40** **nON 3** **nOHNH 0** **nviolations 0** **nrotb 1** **volume 301.64**

#### 3.5.3 Atomic charge calculations and pharmacophore site identification

The identification of the pharmacophore site for each molecule was based on the X and Y atomic charges of each pocket and the corresponding (X-Y) distance. There is a coexistence of two combined antifungal (O1^δ−^---O2^δ−^) and antiviral (O1^δ−^---N1^δ−^) pharmacophore sites, which results in a major issue due to the two-methyl substituents on the central rings (Table 7). For this reason, the potential of this series is likely better as an antiviral than antibacterial and antifungal agents; which requires further experimental validation.

**TABLE 7:** Atomic charge and pharmacophore sites identification of compounds **3a-d**.

Compd	Atomic charge	Pharmacophore sites
**3a**	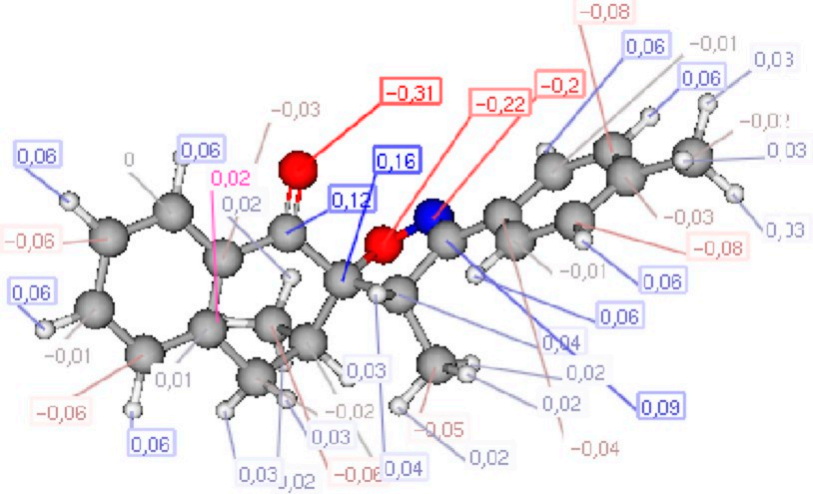	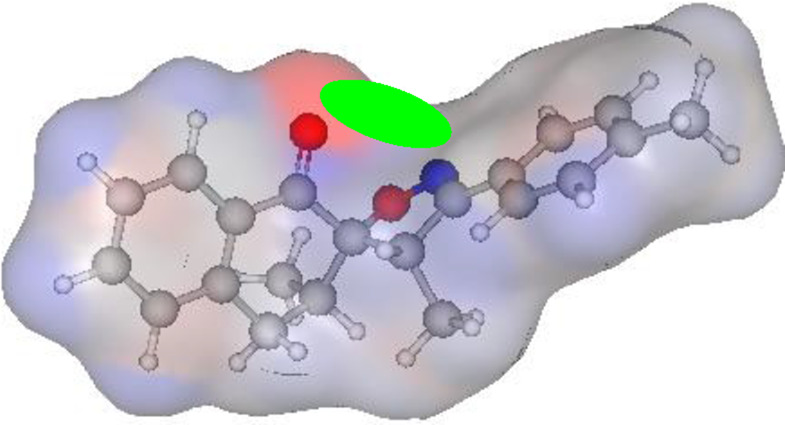
**3b**	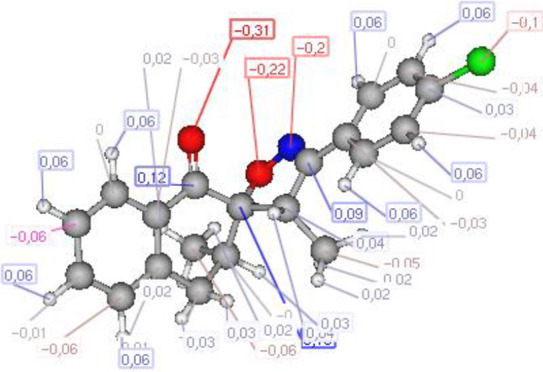	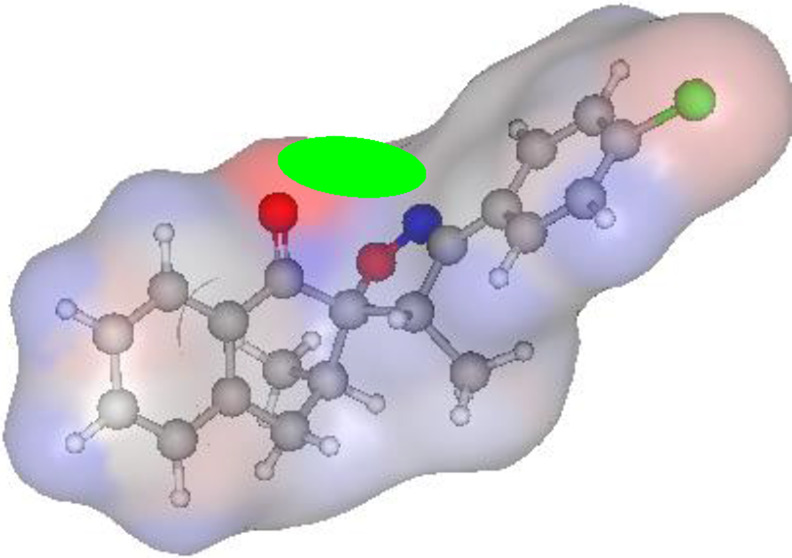
**3c**	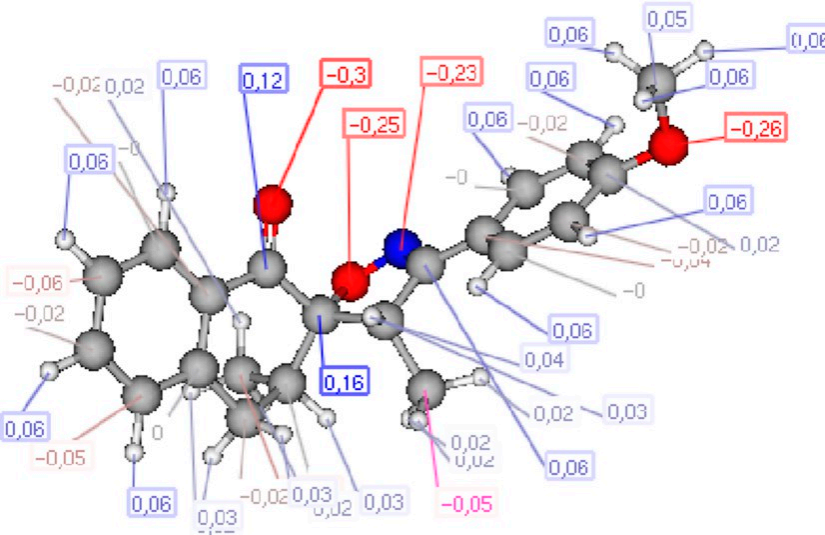	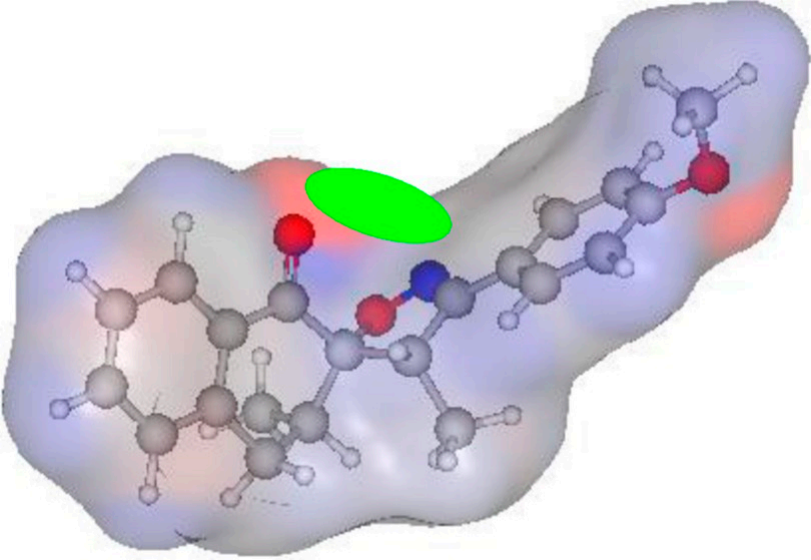
**3d**	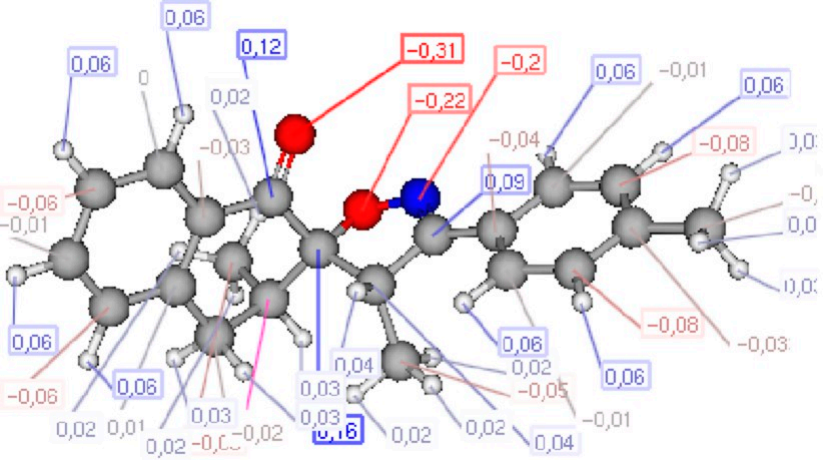	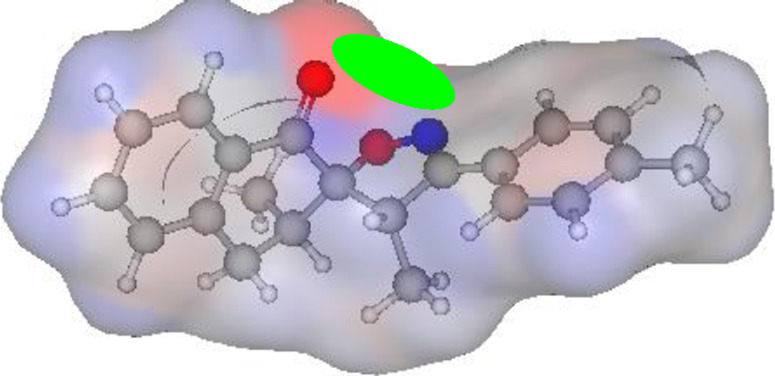
**3a-d**	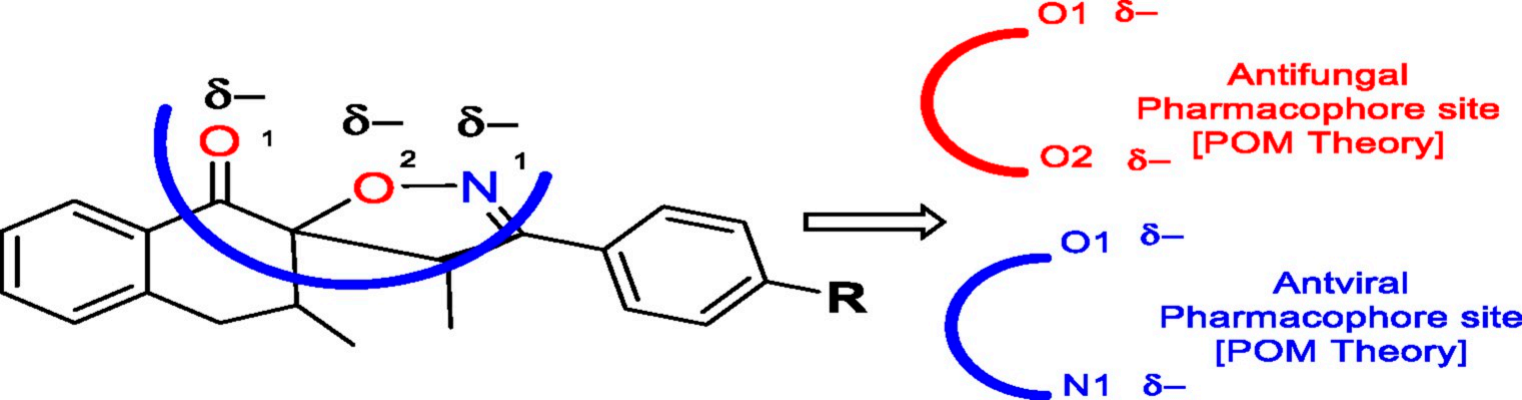 **Common pharmacophore sites of compound 3a-d**	

## 4 Conclusion

A new series of spiroisoxazoline derivatives was synthesized through a regio- and diastereoselective 1,3-DC reaction of the arylnitriloxides as dipoles and (E)-2-ethylidene-3-methyl-3,4-dihydronaphthalene-1(2H)-one as a dipolarophile. The structures and the observed regiochemistry of the synthesized spiroisoxazolines were established using standard spectroscopic methods, and further validated by elemental analysis (EA), and HRMS. Furthermore, mechanistic studies were carried out using DFT calculations with the B3LYP/6-31G (d,p) to gain deeper insight into the regioselective synthesis of new spiro-compounds. The theoretical findings obtained align closely with the experimental observations. The *in vitro* antibacterial screening of the synthesized compounds against a range of bacterial strains, employing agar-well diffusion and microdilution methodologies, revealed that the spiroisoxazoline **3b** demonstrated antimicrobial action against all the pathogenic strains tested, while compound **3c** exhibited antibacterial activity solely against the two Gram-positive bacteria tested. *In silico* studies were also carried out to rationalize the experimental findings and provide mechanistic insight. POM analyses of the relative antimicrobial activity of these derivatives were also performed. Interestingly, drug-likeness analysis suggested that the tested spiro-isoxazolines would require structural optimization to yield derivatives with improved oral bioavailability and favorable brain penetration properties. Therefore, the results of the present investigation suggest that the studied congeners represent promising antiviral candidates, warranting further in-depth investigation. The current study provides important insights into the origins of the modest antimicrobial potential of spiro-compounds, thereby supporting their use as scaffolds in the rational design and development of more potent antiviral drug candidates. Taken together, the combined experimental and theoretical results provide a foundation for the rational design and development of new antiviral spiro-compounds with promising therapeutic potential.

## Data Availability

The original contributions presented in the study are included in the article/Supplementary Material, further inquiries can be directed to the corresponding authors.

## References

[B1] Afsar N. Reuben Jonathan D. Ramesh S. Manivannan S. Ahamed J. I. (2024). Synthesis of biologically active α-Tetralone condensate: growth of single crystal, characterization by experimental and simulation methods. Polycycl. Aromat. Compd. 44, 2394–2418. 10.1080/10406638.2023.2216836

[B2] Ait Assou S. Grafov A. Bousta D. Bekkari H. El Hassouni M. (2024). Unveiling the anti-candida albicans activity of bioactive fractions from mining soil-dwelling streptomyces sp. DS104. Sci. Afr. 25, e02292. 10.1016/j.sciaf.2024.e02292

[B3] Akhazzanea M. Kerbala A. Kella Bennania A. Daoudia M. El Yazidia M. Guarriguesb B. (2011). Reaction de cycloaddition dipolaire-1,3 des arylnitriloxydes vis-a-vis des 4-ethyl-2-[(e)-arylidene]-3,4-dihydro-1(2h) naphtalenones. J. Mar. Chim. Heterocycl.

[B4] AL-Adhreai A. Alsaeedy M. Alrabie A. Al-Qadsy I. Dawbaa S. Alaizeri Z. A. M. (2022). Design and synthesis of novel enantiopure Bis(5-Isoxazolidine) derivatives: insights into their antioxidant and antimicrobial potential via *in silico* drug-likeness, pharmacokinetic, medicinal chemistry properties, and molecular docking studies. Heliyon 8, e09746. 10.1016/j.heliyon.2022.e09746 35800717 PMC9253851

[B5] Al-Maqtari H. M. Jamalis J. Hadda T. B. Sankaranarayanan M. Chander S. Ahmad N. A. (2017). Synthesis, characterization, POM analysis and antifungal activity of novel heterocyclic chalcone derivatives containing acylated pyrazole. SpringerHM Al-Maqtari 43, 1893–1907. 10.1007/S11164-016-2737-Y

[B6] Aljofan M. Netter H. J. Aljarbou A. N. Hadda T. B. Orhan I. E. Sener B. (2014). Anti-hepatitis B activity of isoquinoline alkaloids of plant origin. BA MungallArchives Virology 159, 1119–1128. 10.1007/S00705-013-1937-7 24311152

[B7] Aljohani G. F. El-Hag F. A. A. Bekheit M. S. Ewies E. F. El-Manawaty M. A. (2022). An efficient one-pot synthesis of certain stereoselective Spiro[pyrazole-4,5′-isoxazoline]-5-one derivatives: *in vitro* evaluation of antitumor activities, molecular docking and *in silico* ADME predictions. Chem. Res. Chin. Univ. 38, 1073–1082. 10.1007/s40242-022-1408-3

[B8] Alminderej F. Ghannay S. Omer Elsamani M. Alhawday F. Albadri A. E. A. E. Elbehairi S. E. I. (2023). *In vitro* and *in silico* evaluation of antiproliferative activity of new isoxazolidine derivatives targeting EGFR: design, synthesis, cell cycle analysis, and apoptotic inducers. Pharmaceuticals 16, 1025. 10.3390/PH16071025 37513936 PMC10384175

[B9] Bahsis L. Hrimla M. El Ayouchia H. B. Anane H. Julve M. Stiriba S. E. (2020). 2-aminobenzothiazole-containing copper(II) complex as catalyst in click chemistry: an experimental and theoretical study. Catalysts 10, 776. 10.3390/catal10070776

[B10] Barghady N. Assou S. A. Er-Rajy M. Boujdi K. Arzine A. Rhazi Y. (2024). Design, synthesis, characterization, and theoretical calculations, along with *in silico* and *in vitro* antimicrobial proprieties of new isoxazole-amide conjugates. Open Chem. 22, 20240109. 10.1515/chem-2024-0109

[B11] Bechlem K. Aissaoui M. Belhani B. Rachedi K. O. Bouacida S. Bahadi R. (2020). Synthesis, X-ray crystallographic study and molecular docking of new α-sulfamidophosphonates: POM analyses of their cytotoxic activity. J. Mol. Struct. 1210. 10.1016/J.MOLSTRUC.2020.127990

[B12] Becke A. D. Edgecombe K. E. (1990). A simple measure of electron localization in atomic and molecular systems. J. Chem. Phys. 92, 5397–5403. 10.1063/1.458517

[B13] Bennani B. Kerbal A. Baba B. F. Daoudi M. Warad I. Aljofan M. (2013). Synthesis, characterization, bioactivity, and POM analyses of isothiochromeno[3,4-e] [1,2]oxazines. Med. Chem. Res. 22, 4798–4809. 10.1007/s00044-012-0392-4

[B14] Bouzammit R. Belchkar S. El Fadili M. Kanzouai Y. Aflak N. Chalkha M. (2024a). Synthesis, characterization, DFT mechanistic study, antibacterial activity, molecular modeling, and ADMET properties of novel chromone-isoxazole hybrids. J. Mol. Struct. 1314, 138770. 10.1016/J.MOLSTRUC.2024.138770

[B15] Bouzammit R. Belchkar S. El fadili M. Kanzouai Y. Mujwar S. Alanazi M. M. (2024b). New triazole-isoxazole hybrids as antibacterial agents: design, synthesis, characterization, *in vitro,* and *in silico* studies. Molecules 29, 2510. 10.3390/MOLECULES29112510 38893386 PMC11174038

[B16] Bouzammit R. Lakkab I. El fadili M. Kanzouai Y. Chalkha M. Nakkabi A. (2024c). Synthesis, crystal structure, antioxidant activity and molecular docking studies of 2-(1-(3-methyl-1-oxo-1,2,3,4-tetrahydronaphthalen-2-yl)ethyl)malononitrile. J. Mol. Struct. 1312, 138582. 10.1016/j.molstruc.2024.138582

[B17] Bouzammit R. El Hachlafi N. El fadili M. El Khabchi M. Kanzouai Y. Salghi R. (2025). Synthesis, crystal structure, DFT calculations, *in-vitro* and *in-silico* studies of novel chromone-isoxazoline conjugates as antibacterial and anti-inflammatory agents. Sci. Rep. 15, 31103–31125. 10.1038/S41598-025-11182-9 40851012 PMC12375725

[B18] Cao M. H. Green N. J. Xu S. Z. (2017). Application of the aza-Diels–Alder reaction in the synthesis of natural products. Org. Biomol. Chem. 15, 3105–3129. 10.1039/C6OB02761J 28327756

[B19] Carnovali M. Ciavatta M. L. Mollo E. Roussis V. Banfi G. Carbone M. (2022). Aerophobin-1 from the marine sponge Aplysina aerophoba modulates osteogenesis in zebrafish larvae. Mar. Drugs 20, 135. 10.3390/md20020135 35200664 PMC8880152

[B20] Chalkha M. Nour H. Chebbac K. Nakkabi A. Bahsis L. Bakhouch M. (2022). Synthesis, characterization, DFT mechanistic study, antimicrobial activity, molecular modeling, and ADMET properties of novel pyrazole-isoxazoline hybrids. ACS Omega 7, 46731–46744. 10.1021/acsomega.2c05788 36570248 PMC9773794

[B21] Chattaraj P. K. Sarkar U. Roy D. R. (2006). Electrophilicity index. Chem. Rev. 106, 2065–2091. 10.1021/cr040109f 16771443

[B22] Dai J. Parrish S. Yoshida W. Yip M. R. Turkson J. Kelly M. (2016). Bromotyrosine-derived metabolites from an Indonesian marine sponge in the family aplysinellidae (order Verongiida). P WilliamsBioorganic & Medicinal Chemistry Letters 26, 499–504. 10.1016/j.bmcl.2015.11.086 26711149 PMC4706807

[B23] Das P. Hasan M. H. Mitra D. Bollavarapu R. Valente E. J. Tandon R. (2019). Design, synthesis, and preliminary studies of spiro-isoxazoline-peroxides against human cytomegalovirus and glioblastoma. J. Org. Chem. 84, 6992–7006. 10.1021/acs.joc.9b00746 31066280

[B24] Das P. Boone S. Mitra D. Turner L. Tandon R. Raucher D. (2020). Synthesis and biological evaluation of fluoro-substituted spiro-isoxazolines as potential anti-viral and anti-cancer agents. RSC Adv. 10, 30223–30237. 10.1039/d0ra06148d 35518245 PMC9056317

[B25] Domingo L. R. (2014). A new C-C bond formation model based on the quantum chemical topology of electron density. RSC Adv. 4, 32415–32428. 10.1039/c4ra04280h

[B26] Domingo L. R. (2016). Molecular electron density theory: a modern view of reactivity in organic chemistry. Molecules 21, 1–15. 10.3390/molecules21101319 27706053 PMC6273663

[B27] Domingo L. R. Ríos-Gutiérrez M. (2017). A molecular electron density theory study of the reactivity of azomethine imine in [3+2] cycloaddition reactions. Molecules 22, 750. 10.3390/molecules22050750 28481228 PMC6154604

[B28] Domingo L. R. Aurell M. J. Pérez P. Contreras R. (2002). Quantitative characterization of the global electrophilicity power of common diene/dienophile pairs in diels–alder reactions. Tetrahedron 58, 4417–4423. 10.1016/S0040-4020(02)00410-6

[B29] Domingo L. R. Pérez P. Sáez J. A. (2013). Understanding the local reactivity in polar organic reactions through electrophilic and nucleophilic parr functions. RSC Adv. 3, 1486–1494. 10.1039/C2RA22886F

[B30] Domingo L. R. Ríos-Gutiérrez M. Aurell M. J. (2021). Unveiling the regioselectivity in electrophilic aromatic substitution reactions of deactivated benzenes through molecular electron density theory. New J. Chem. 45, 13626–13638. 10.1039/D1NJ02435C

[B31] Domingo L. R. Ríos-Gutiérrez M. Acharjee N. (2022). A molecular electron density theory study of the lewis acid catalyzed [3+2] cycloaddition reactions of nitrones with nucleophilic ethylenes. Eur. J. Org. Chem., 2022. 10.1002/EJOC.202101417

[B32] Ech-chihbi E. Bouzammit R. Salim R. Er-rajy M. Salghi R. Azgaou K. (2025). Chromone-isoxazole derivatives as corrosion inhibitors for mild steel in 1 M HCl solution: experimental, DFT and DFTB approaches. Colloids Surf. A Physicochem Eng. Asp. 720, 137169. 10.1016/J.COLSURFA.2025.137169

[B82] Er-rajy M. El fadili M. Mujwar S. Zarougui S. Elhallaoui M. (2023). Design of novel anti-cancer drugs targeting TRKs inhibitors based 3D QSAR, molecular docking and molecular dynamics simulation. J. Biomol. Struct. Dyn. 41, 11657–11670. 10.1080/07391102.2023.2170471 36695085

[B33] Er-Rajy M. Faris A. Zarougui S. Anti-Cancer M. E. (2024). Design of potential anti-cancer agents as COX-2 inhibitors, using 3D-QSAR modeling, molecular docking, oral bioavailability proprieties, and molecular dynamics. journals.lww.comM Er-Rajy. 10.1097/CAD.0000000000001578 38018861

[B34] Er-rajy M. El fadili M. Zarougui S. Mujwar S. Aloui M. Zerrouk M. (2025). Design and evaluation of novel triazole derivatives as potential anti-gout inhibitors: a comprehensive molecular modeling study. Front. Chem. 13, 1518777. 10.3389/fchem.2025.1518777 40115054 PMC11922854

[B35] Ferreira Montenegro P. Pham G. N. Abdoul-Latif F. M. Taffin-de-Givenchy E. Mehiri M. (2024). Marine bromotyrosine derivatives in spotlight: bringing discoveries and biological significance. Mar. Drugs 22, 132. 10.3390/md22030132 38535473 PMC10971822

[B36] Fihi R. Ciamala K. Vebrel J. Rodier N. (1995). Reaction des methylene γ-butyrolactones avec les arylnitriloxydes. evolution inattendue du bisadduit issu de la 5-methylene(5h)furan-2-one. Bull. Des. Sociétés Chim. Belg. 104, 55–62. 10.1002/BSCB.19951040110

[B37] Frisch M. J. Trucks G. W. Schlegel H. B. Scuseria G. E. Robb M. A. Cheeseman J. R. (2009). Gaussian 09. Wallingford CT: Gaussian Inc.

[B38] Grib I. Berredjem M. Rachedi K. O. Djouad S. E. Bouacida S. Bahadi R. (2020). Novel N-sulfonylphthalimides: efficient synthesis, X-ray characterization, spectral investigations, POM analyses, DFT computations and antibacterial activity. Elsevier, 1217. 10.1016/J.MOLSTRUC.2020.128423

[B39] Grundmann C. Richter R. (1968). Nitrile oxides. X. Improved method for the prepared of nitrile oxides from aldoximes. J. Org. Chem. 33, 476–478. 10.1021/jo01265a120

[B40] Guo Y. Zhang Q. Liu Z. Bao C. Fan J. Yang R. (2019). Non-food bioactive products: design and semisynthesis of novel (+)-nootkatone derivatives containing isoxazoline moiety as insecticide candidates. Ind. Crops Prod. 140, 111706. 10.1016/J.INDCROP.2019.111706

[B41] Hockett K. L. Baltrus D. A. (2017). Use of the soft-agar overlay technique to screen for bacterially produced inhibitory compounds. J. Vis. Exp. 2017, 1–5. 10.3791/55064 28117830 PMC5352255

[B42] Hong L. Wang R. (2013). Recent advances in asymmetric organocatalytic construction of 3,3′-Spirocyclic oxindoles. Adv. Synth. Catal. 355, 1023–1052. 10.1002/ADSC.201200808

[B43] Jarrahpour A. Heiran R. Sinou V. Latour C. Djouhri Bouktab L. Michel Brunel J. (2019). Synthesis of new β-lactams bearing the biologically important morpholine ring and POM analyses of their antimicrobial and antimalarial activities. Iran. J. Pharm. Res 18 (1), 34–48. 31089342 PMC6487420

[B44] Kalhor H. Movahhed T. K. Mousavi S. Qomi M. S. Abolhasani A. Mirani M. (2024). Anti-cancer activity evaluation of naphthalenonic and chromanonic spiroisoxazoline derivatives as selective COX-2 inhibitors on HT29 cell lines. Clin. Cancer Drugs 10, E110724231864. 10.2174/012212697X274833240408033609

[B45] Kanzouai Y. Chalkha M. Hadni H. Laghmari M. Bouzammit R. Nakkabi A. (2023). Design, synthesis, *in-vitro* and *in-silico* studies of chromone-isoxazoline conjugates as anti-bacterial agents. J. Mol. Struct. 1293, 136205. 10.1016/J.MOLSTRUC.2023.136205

[B46] Kaur M. Singh B. Singh B. Arjuna A. (2017). Synthesis and evaluation of novel spiro[oxindole-isoxazolidine] derivatives as potent antioxidants. J. Heterocycl. Chem. 54, 1348–1354. 10.1002/JHET.2712

[B47] Kawsar S. M. A. Hosen M. A. Ahmad S. El Bakri Y. Laaroussi H. Hadda T. B. (2022). Potential SARS-CoV-2 RdRp inhibitors of cytidine derivatives: molecular docking, molecular dynamic simulations, ADMET, and POM analyses for the identification of pharmacophore sites. PLoS One 17, e0273256. 10.1371/JOURNAL.PONE.0273256 36441684 PMC9704642

[B48] Khodair A. I. El-Barbary A. A. Imam D. R. Kheder N. A. Elmalki F. Ben Hadda T. (2021). Synthesis, antiviral, DFT and molecular docking studies of some novel 1, 2, 4-triazine nucleosides as potential bioactive compounds. Elsevier, 500. 10.1016/j.carres.2021.108246 33516074

[B49] Kumar G. Tabassum M. Sharma B. K. Kumar R. Tali J. A. Singh D. (2024). Design and synthesis of C-8 spiro-isoxazoline analogues of 14-Deoxy-11,12-didehydroandrographolide (14-DDA) for dual targeting of CDK4 and BCL2 mediated anticancer activity. J. Mol. Struct. 1298, 137072. 10.1016/j.molstruc.2023.137072

[B50] Lakhrissi Y. Rbaa M. Tuzun B. (2022). Synthesis, structural confirmation, antibacterial properties and bio-informatics computational analyses of new pyrrole based on 8-hydroxyquinoline. Elsevier. Available online at: https://www.sciencedirect.com/science/article/pii/S0022286022003568 (Accessed October 8, 2025).

[B51] Lee C. Yang W. Parr R. G. (1988). Development of the colle-salvetti correlation-energy formula into a functional of the electron density. Phys. Rev. B 37, 785–789. 10.1103/PhysRevB.37.785 9944570

[B52] Lu T. Chen F. (2012). Multiwfn: a multifunctional wavefunction analyzer. J. Comput. Chem. 33, 580–592. 10.1002/JCC.22885 22162017

[B53] Mabkhot Y. N. Barakat A. Yousuf S. Choudhary M. I. Frey W. Ben Hadda T. (2014). Substituted thieno[2,3-b]thiophenes and related congeners: Synthesis, β-glucuronidase inhibition activity, crystal structure, and POM analyses. Med. Chem. 22, 6715–6725. 10.1016/J.BMC.2014.08.014 25245672

[B83] Madadi Mahani N. Hamidian H. Fozooni S. Salajeghe M. (2025). Synthesis, TD-DFT calculations, molecular docking and ADME studies of new spiro-oxindole derivatives containing 5(4H)-oxazolone as anti-viral and anti-bacterial agents. J. Comput.-Aided Mol. Des. 39. 10.1007/s10822-025-00593-5 40214849

[B54] Mane S. G. Reddy D. S. Katagi K. S. Kumar A. Munnolli R. S. Kadam N. S. (2021). Design, synthesis, molecular docking, anti-proliferative and anti-TB studies of 2H-chromen-8-azaspiro[4.5]decane-7,9-dione conjugates. J. Mol. Struct. 1227, 129530. 10.1016/J.MOLSTRUC.2020.129530

[B55] Martins L. O. Soares C. M. Pereira M. M. Teixeira M. Costa T. Jones G. H. (2002). Molecular and biochemical characterization of a highly stable bacterial laccase that occurs as a structural component of the Bacillus subtilis endospore coat. jbc.Org. 277, 18849–18859. 10.1074/JBC.M200827200 11884407

[B56] Mezgebe K. Mulugeta E. (2022). Synthesis and pharmacological activities of azo dye derivatives incorporating heterocyclic scaffolds: a review. RSC Adv. 12, 25932–25946. 10.1039/D2RA04934A 36199603 PMC9469491

[B57] Mills N. (2006). ChemDraw ultra 10.0 CambridgeSoft, 100 CambridgePark drive. J. Am. Chem. Soc. 128, 02140. 10.1021/JA0697875

[B58] Moriou C. Lacroix D. Petek S. El-Demerdash A. Trepos R. Leu T. M. (2021). Bioactive bromotyrosine derivatives from the Pacific marine sponge Suberea clavata (Pulitzer-Finali, 1982). Mar. Drugs 19, 1–22. 10.3390/MD19030143 33800819 PMC7999702

[B59] Najim N. Bathich Y. Zain M. M. Hamzah A. S. Shaameri Z. (2010). Evaluation of the bioactivity of novel spiroisoxazoline TypeCompounds against normal and cancer cell lines. Molecules 15, 9340–9353. 10.3390/MOLECULES15129340 21169884 PMC6259157

[B60] Paciorek J. Höfler D. Sokol K. R. Wurst K. Magauer T. (2022). Total synthesis of the dihydrooxepine-spiroisoxazoline natural product psammaplysin A. J. Am. Chem. Soc. 144, 19704–19708. 10.1021/jacs.2c10010 36270001 PMC9634798

[B61] Pfaller M. A. Haturvedi V. Espinel-Ingroff A. Ghannoum M. A. Gosey L. L. Odds F. C. (2002). Reference method for broth dilution antifungal susceptibility testing of yeasts; approved standard — second edition serving the world ’ s medical science community through voluntary consensus.

[B62] Pratap S. Naaz F. Reddy S. Jha K. K. Sharma K. Sahal D. (2019). Anti-proliferative and anti-malarial activities of spiroisoxazoline analogues of artemisinin. Arch. Pharm. Weinh. 352, 1800192. 10.1002/ARDP.201800192 30537298

[B63] Rachedi K. O. Bahadi R. Aissaoui M. Hadda T. B. Belhani B. Bouzina A. (2020). DFT study, POM analyses and molecular docking of novel oxazaphosphinanes: identification of antifungal pharmacophore site. J. ugm.ac 20, 440–450. 10.22146/IJC.46375

[B64] Raju M. Hemasri Y. Rao Y. J. (2024). Synthesis of new class of spirochromanone/isoxazole/isoxazoline derivatives as potential antimicrobial agents. Russ. J. Gen. Chem. 94, 2460–2469. 10.1134/s107036322409024x

[B65] Rana R. Ansari A. (2023). Synthesis of 7-Membered heterocyclic compounds and their biological activity. J. Phys. Conf. Ser. 2603, 012058. 10.1088/1742-6596/2603/1/012058

[B66] Rani D. Garg V. Dutt R. (2021). Anticancer potential of azole containing marine natural products: current and future perspectives. Anticancer Agents Med. Chem. 21, 1957–1976. 10.2174/1871520621666210112112422 33438564

[B67] Rbaa M. Jabli S. Lakhrissi Y. Ouhssine M. Almalki F. Ben Hadda T. (2019). Synthesis, antibacterial properties and bioinformatics computational analyses of novel 8-hydroxyquinoline derivatives. Cell 5, e02689. 10.1016/J.HELIYON.2019.E02689 31687516 PMC6820249

[B68] Ríos-Gutiérrez M. Domingo L. R. (2019a). The carbenoid-type reactivity of simplest nitrile imine from a molecular electron density theory perspective. Tetrahedron 75, 1961–1967. 10.1016/j.tet.2019.02.014

[B69] Ríos-Gutiérrez M. Domingo L. R. (2019b). Unravelling the mysteries of the [3+2] cycloaddition reactions. Eur. J. Org. Chem. 2019, 267–282. 10.1002/EJOC.201800916

[B70] Ríos-Gutiérrez M. Domingo L. Ghodsi F. (2021). Unveiling the different reactivity of bent and linear three-atom-components participating in [3 + 2] cycloaddition reactions. Organics 2, 274–286. 10.3390/org2030014

[B71] Sawhney G. Rasool J. U. Saroch D. Ozturk M. Brombacher F. Ahmad B. (2022). Arteannuin-B and (3-Chlorophenyl)-2-Spiroisoxazoline derivative exhibit anti-inflammatory effects in LPS-activated RAW 264.7 macrophages and BALB/c mice-induced proinflammatory responses via downregulation of NF-κB/P38 MAPK signaling. Molecules 27, 8068. 10.3390/MOLECULES27228068 36432169 PMC9699497

[B72] Shinde Y. Khairnar B. Bangale S. (2024). Exploring the diverse biological frontiers of isoxazole: a comprehensive review of its pharmacological significance. ChemistrySelect 9, e202401423. 10.1002/SLCT.202401423

[B73] Systèmes D. (2024). Free download: BIOVIA discovery studio visualizer. Dassault Systèmes. Available online at: https://discover.3ds.com/discovery-studio-visualizer-download.

[B74] Tóth G. Balázs B. Lévai A. Fišera L. Jedlovská E. (1999). Stereochemistry of some spiropyrazolines and spiroisoxazolines. J. Mol. Struct. 508, 29–36. 10.1016/S0022-2860(98)00912-0

[B75] Trott O. Olson A. J. (2010). AutoDock vina: improving the speed and accuracy of docking with a new scoring function, efficient optimization, and multithreading. J. Comput. Chem. 31 (2), 455–461. 10.1002/jcc.21334 19499576 PMC3041641

[B76] Tu L. Gao L. Wang Q. Cao Z. Huang R. Zheng Y. (2022). Substrate-switched chemodivergent pyrazole and pyrazoline synthesis: [3 + 2] Cycloaddition/ring-opening rearrangement reaction of azadienes with nitrile imines. J. Org. Chem. 87, 3389–3401. 10.1021/acs.joc.1c02998 35157462

[B77] Ukaji Y. Soeta T. (2014). Development of new methods for the construction of heterocycles based on cycloaddition reaction of 1,3-Dipoles. 263–282. 10.1002/9781118778173.CH11

[B78] Uppadhayay R. K. Kumar A. Teotia J. Singh A. (2022). Multifaceted chemistry of tetrazole. Synthesis, uses, and pharmaceutical applications. Russ. J. Org. Chem. 58, 1801–1811. 10.1134/s1070428022120090

[B79] Wang S. Yuan X. H. Wang S. Q. Zhao W. Chen X. B. Yu B. (2021). FDA-Approved pyrimidine-fused bicyclic heterocycles for cancer therapy: synthesis and clinical application. Eur. J. Med. Chem. 214, 113218. 10.1016/J.EJMECH.2021.113218 33540357

[B80] Wang X. Hu Q. Tang H. Pan X. (2023). Isoxazole/isoxazoline skeleton in the structural modification of natural products: a review. Pharmaceuticals 16, 228. 10.3390/ph16020228 37259376 PMC9964809

[B81] Yoshida H. Kawai F. Obayashi E. Akashi S. Roper D. I. Tame J. R. H. (2012). Crystal structures of penicillin-binding protein 3 (PBP3) from methicillin-resistant *Staphylococcus aureus* in the Apo and cefotaxime‐bound forms. SY Park. Molecular Biology 423, 351–364. 10.1016/J.JMB.2012.07.012 22846910

