# Supplementary material for: Regioselective synthesis of novel spiro-isoxazolines congeners as antimicrobial agents: in vitro and in-silico assessments
Source: Front Chem. 2025 Dec 29;13:1740409. doi: 10.3389/fchem.2025.1740409 (PMC12791170; doi:10.3389/fchem.2025.1740409)
Supplement: Supplementary file 1 [file DataSheet1.docx]

**Regioselective Synthesis of Novel Spiro-Isoxazolines Congeners as antimicrobial agents: *In Vitro* and *In-silico* Assessments**

Rachid BOUZAMMIT ^a^, Soumia AIT ASSOU ^b^, Mohammed ER-RAJY ^c^, Noura AFLAK ^d^, Lahoucine BAHSIS ^e^, Mohammed CHALKHA ^a,f,*^, Mohammed El HASSOUNI ^b^, Mohammed LACHKAR ^a^, Taibi BEN HADDA ^g^ , Daryn BENSON ^g^ Abdullah A. ALYOUSEF^h^, Mourad A. M. ABOUL-SOUD^i^, John P. GIESY^j,k,l^ and Ghali AL HOUARI ^a,*^

*^a^Engineering Laboratory of Organometallic, Molecular Materials and Environment (LIMOME), Faculty of Sciences, University Sidi Mohamed Ben Abdellah, 30000 Fez, Morocco.*

*^b^Biotechnology, Environment, Agri-Food and Health Laboratory, Faculty of Sciences Dhar El Mahraz, Sidi Mohamed Ben Abdellah University, BP. 1796, Atlas, Fez, Morocco.*

*^c^LIMAS Laboratory, Chemistry Department, Faculty of Sciences Dhar El Mahraz, Sidi Mohamed Ben Abdellah University, 30000 Fez, Morocco.*

*^d^Team of Organic Chemistry and Valorization of Natural Substances, Faculty of Sciences, University Ibn Zohr, BP 8106, Cite Dakhla, 80000 Agadir, Morocco.*

*^e^Faculté Polydisciplinaire de Safi, Université Cadi Ayyad, 46030 Safi, Morocco.*

*^f^Laboratory of Materials Engineering for the Environment and Natural Resources, Faculty of Sciences and Techniques, University of Moulay Ismail, B.P 509, Boutalamine, 52000, Errachidia, Morocco.*

*^g^Euromed University of Fes (UEMF), Road of Meknez, Fez 30000, Morocco.*

*^h^ Clinical Laboratory Sciences Department, College of Applied Medical Sciences, King Saud University, P.O. Box 17 10219, Riyadh 11433, Saudi Arabia*

*^i^Center of Excellence in Biotechnology Research (CEBR), College of Applied Medical Sciences, King Saud University, P.O. Box 17 10219, Riyadh 11433, Saudi Arabia*

*^j^Department of Veterinary Biomedical Sciences and Toxicology Centre, Western College of Veterinary Medicine, University of Saskatchewan, Saskatoon, SK S7N 5B4, Canada,*

*^k^Department of Integrative Biology and Center for Integrative Toxicology, Michigan State University, East Lansing, MI 48824, USA*

*^l^Department of Environmental Sciences, Baylor University, Waco, 76706, USA*

*Corresponding authors: Mohammed CHALKHA (mohammed.chalkha1@usmba.ac.ma), Ghali AL HOUARI (ghalialhouari@gmail.com )

1. **General information**

All the chemicals used were of analytical grade and were employed without further purification, sourced from commercial suppliers. The progress of the reactions was monitored by thin-layer chromatography (TLC) (Merck, silica gel 60 F254), with spots visualized under UV light (Vilber Lourmat, VL-215.LC). The melting points were determined with an uncertainty of ±2 °C using a Koﬂer Bench. The IR spectra were recorded in the range of 4000 to 450 cm⁻¹ on a Bruker Vertex 70 FT-IR spectrometer, and wavenumbers are given in cm⁻¹. The NMR spectra (^1^H and ^13^C) were recorded at room temperature on a Bruker Avance II 300 UltraShield spectrometer (300 MHz for ^1^H and 75 MHz for ^13^C) using CDCl_3_ as the solvent. For the ^13^C NMR spectra, the APT experiment was used, providing information on the multiplicity of the ^13^C signals (CH_3_, CH_2_, CH, and Cq). In these spectra, the negative signals correspond to the CH_3_ and CH carbons, while the positive signals correspond to the CH_2_ and quaternary C carbons. The chemical shifts are expressed in ppm and the coupling constants J in Hertz (Hz). The spin multiplicities are reported as singlet (s), doublet (d), triplet (t), multiplet (m), doublet of doublets (dd), doublet of triplets (dt), and broad (br). High-resolution mass spectra were recorded on a Waters/Vion IMS-QTOF spectrometer, equipped with an electrospray ionization (ESI) source operating in both positive and negative ion modes.

1. **Method for synthesizing spiroisoxazolines**

In a 100 ml Erlenmeyer flask, introduce 2 mmol of arylidene tetralone 1 and 2.4 mmol of aryl nitrile oxide 2. Dissolve these reactants in 15 ml of chloroform (CHCl₃), then add five drops of trimethylamine (Et₃N). Stir the mixture using a magnetic stirrer at room temperature (20-30 °C) for 34 hours to ensure the reaction is complete. The reaction progress is monitored periodically by thin-layer chromatography (TLC) using an appropriate eluent system (7/3: hexane/ether). Once the reaction is complete, transfer the mixture to a separating funnel and wash until a pH of 7 is achieved. Dry the product over anhydrous sodium sulfate and filter. Evaporate the chloroform from the organic layer under reduced pressure using a rotary evaporator. Finally, recrystallize the obtained solid in a mixture of ethanol/dichloromethane (8/2: EtOH/CH_2_Cl_2_) and collect the pure solids by vacuum filtration.

- (**3a**): 3',4-dimethyl-3-phenyl-3',4'-dihydro-1'H,4H-spiro[isoxazole-5,2'-naphthalene]-1'-one.

Orange crystals, m.p = 168-170 ºC, Yield 68%; **IR** (KBr, ν in cm^-1^): ν =1700 (C=O); **^1^H NMR** (300 MHz, CDCl_3_, δ in ppm): δ =1.11 (d, 3H, CH_3(3’’)_, *J* = 7.2 Hz ), 1.30 (d, 3H, CH_3(4’’)_, *J* = 7.2 Hz), 2.78-3.80 (dd, 2H_4,_ CH_2_, *J* = 7.2 Hz), 2.90 (m, H_3_), 4.31 (q, H**_4’iso_**, *J*= 7.2 Hz), 7.26-7.35 (m, 2H-Ar), 7.42-7.47 (m, 3H-Ar), 7.53 (tt, 1H-Ar, *J* = 1.5 Hz, *J* = 6 Hz, *J* = 7.5 Hz), 7.78-7.98 (m, 2H-Ar), 7.96 (dd, 1H-Ar, *J* = 1.2 Hz, *J* = 6.6 Hz); **^13^C NMR** (75 MHz, CDCl_3,_ δ in ppm): CH_3(3’’)_ (11.54), CH_3(4’’)_ (15.40), CH**_4’iso_** (32.05), C_3_ (40.65), C_4_ (33.32), 40.65, C**_2,5’_** (90.81), 126.67,127.37, 128.13, 128.41, 128.84, 129.41, 130.30, 130.80, 133.88, 141.12, 164.78, C**_3’_**=N (164.78), C=O (192.04); **Elementary analysis**: (calculated /found) %C = 78.68/78.0103, %H = 6.22/6.2251, %N = 4.59/4.5427; **ESI-QTOF-MS** (m/z): mass calculated for [C_20_H_19_NO_2_-H]^+^: 306.14886, mass found: 306.14850.

- (**3b):** 3-(4-chlorophényl)-3',4-diméthyl-3',4'-dihydro-1'H,4H-spiro[isoxazole-5,2'-naphtalène]-1'-one.

White crystals, m.p = 170 ºC, Yield 84%; **IR** (KBr, ν in cm^-1^): ν =1690 (C=O); **^1^H NMR** (300 MHz, CDCl_3_, δ in ppm): δ =1.00 (d, 3H, CH_3(3’’)_, *J* = 6.9 Hz), 1.18 (d, 3H, CH_3(4’’)_, *J* = 7.2 Hz), 2.81-2.92 (m, 1H_4_, 1H_3_), 3.53 (dd, 1H_4_, *J* = 4.5 Hz, *J* = 12.6 Hz), 4.29 (q, H**_4’iso_,** *J* = 7.2 Hz), 7.36-7.41 (m, 2H-Ar), 7.56-7.66 (m, 3H-Ar), 7.79-7.87 (m, 3H-Ar); **^13^C NMR** (75 MHz, CDCl_3,_ δ in ppm): CH_3(3’’)_ (11.74), CH_3(4’’)_ (15.34), CH**_4’iso_** (31.51), C_4_ (39.16), C_3_ (40.00), C**_2,5_’** (91.21), 127.27, 127.32, 127.66, 129.26, 129.70, 130.28, 130.52, 134.78, 135.65, 141.62, C**_3’_**=N (164.34), C=O (191.71); **Elementary.analysis** :.( calculated/found) %C=70.76/70.1565, %H=5.30/5.3095, %N=4.12/4.3207; **ESI-QTOF-MS** (m/z): mass calculated for [C_20_H_18_ClNO_2_-H]^+^: 340.10988, mass found: 340.10954.

- (**3c):** 3-(4-methoxyphenyl)-3',4-dimethyl-3',4'-dihydro-1'H,4H-spiro[isoxazole-5,2'-naphthalene]-1'-one.

Orange crystals, m.p **=** 208 ºC, Rdt 88%; **IR** (KBr, ν in cm^-1^): ν =1690 (C=O); **^1^H NMR** (300 MHz, CDCl_3_, δ in ppm); δ =1.10 (d, 3H, CH_3(3’’)_, *J* = 6.9 Hz), 1.29 (d, 3H, CH_3(4’’)_, *J* = 7.2 Hz), 2.77-3.78 (dd, 2H_4,_ CH_2_, *J* = 1.5 Hz, *J* = 15.6 Hz), 2.90-2.95 (m, H_3_), 3.96 (s, Ar-OCH_3_), 4.22 (q, H**_4’iso_,** *J* = 7.5 Hz), 6.79 (d, H-Ar, *J* = 8.4 Hz), 7.00-7.34 (m, 3H-Ar), 7.49-7.55 (m, 1H-Ar), 7.65-7.68 (dd, 1H-Ar, *J* = 2.1 Hz, *J* = 6.6 Hz), 7.82 (d, 1H-Ar, *J* = 2.1 Hz), 7.99 (dd, 1H-Ar, *J* = 0.9 Hz, *J* = 6.9 Hz), **^13^C NMR** (75 MHz, CDCl_3,_ δ in ppm): CH_3(3’’)_ (11.53), CH_3(4’’)_ (15.35), CH**_4’iso_** (31.99), C_4_ (33.29), C_3_ (40.72), Ar-OCH_3_ (56.29), C**_2,5’_** (90.83), 12.05, 121.85, 123.06, 126.68, 127.07, 128.11, 129.16, 129.41, 130.73, 133.91,141.09, C**_3’_**=N (163.51), C=O (191.98); **Elementary.analysis**: (calculated/found) %C=75.13/74.8636, %H=6.26/5.6851, %N=4.17/3.7915; **ESI-QTOF-MS** (m/z): mass calculated for [C_21_H_21_NO_3_-H]^+^: 336.15942, mass found: 336.15905.

- (**3d):** 3',4-dimethyl-3-(p-tolyl)-3',4'-dihydro-1'H,4H-spiro[isoxazole-5,2'-naphthalene]-1'-one.

White crystals, m.p = 198-200 ºC, Yield 63%; **IR** (KBr, ν in cm^-1^): ν =1690 (C=O); **^1^H NMR** (300 MHz, CDCl_3_, δ in ppm): δ =1.10 (d, 3H, CH_3(3’’)_, *J* = 7.2 Hz), 1.30 (d, 3H, CH_3(4’’)_, *J* = 7.5 Hz), 2.41 (s, Ar-CH_3_), 2.78-3.79 (dd, 2H_4,_ CH_2_, *J* = 1.5 Hz, *J* = 15.6 Hz), 2.90-2.94 (m, H_3_), 4.29 (q, H**_4’iso_**, *J* = 7.5 Hz), 7.24-7.49 (m, 3H-Ar), 7.52 (tt, 1H-Ar, *J* = 1.5 Hz, *J* = 6 Hz, *J* = 7.5 Hz), 7.70 (d, 2H-Ar), 7.67 (dd, 1H-Ar, *J* = 1.2 Hz, *J* = 6.6 Hz); **^13^C NMR** (75 MHz, CDCl_3,_ δ in ppm): CH_3(3’’)_ (11.59), CH_3(4’’)_ (15.40), Ar-CH_3_ (21.50), CH**_4’iso_** (32.06), C_4_ (33.33), C_3_ (40.65), C**_2,5’_** (90.61), 125.55, 126.64,127.31, 128.12, 129.39, 129.54, 130.84, 133.82, 140.55, 141.11, C**_3’_**=N (164.74), C=O (192.11); **Elementary.analysis**:.( calculated/found).%C=78.99/78.4700, %H=6.58/6.6016; %N=4.38/4.3599; **ESI-QTOF-MS** (m/z): mass calculated for [C_21_H_21_NO_2_-H]^+^: 320.16451, mass found: 320.16395.

1. **Copies of IR, ^1^H, ^13^C NMR and HRMS spectra of compounds**

## Spectra of compound **3a-d**

## Spectra of 3',4-dimethyl-3-phenyl-3',4'-dihydro-1'H,4H-spiro[isoxazole-5,2' naphthalen]-1'-one **3a**


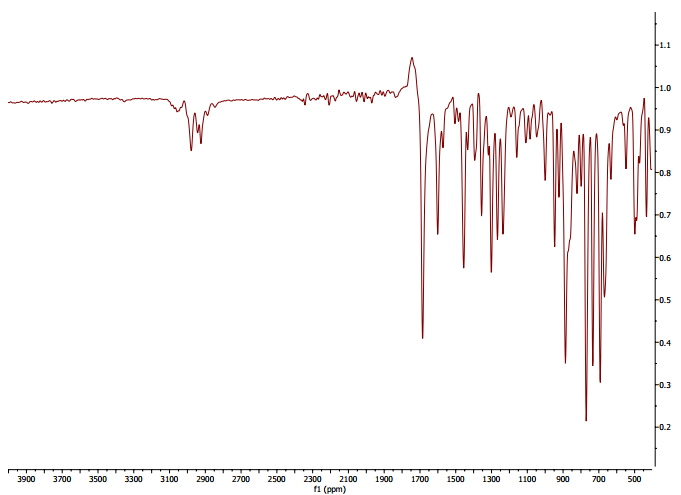


Figure 1. IR spectra of compound 3a


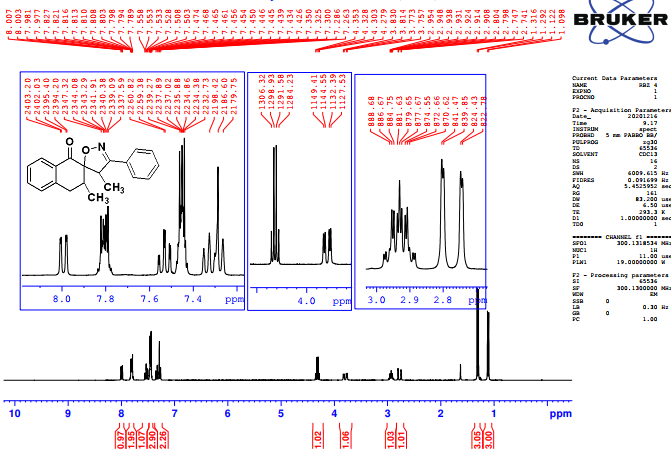


Figure 2. ^1^H NMR spectrum (300 MHz, CDCl_3_) of compound 3a


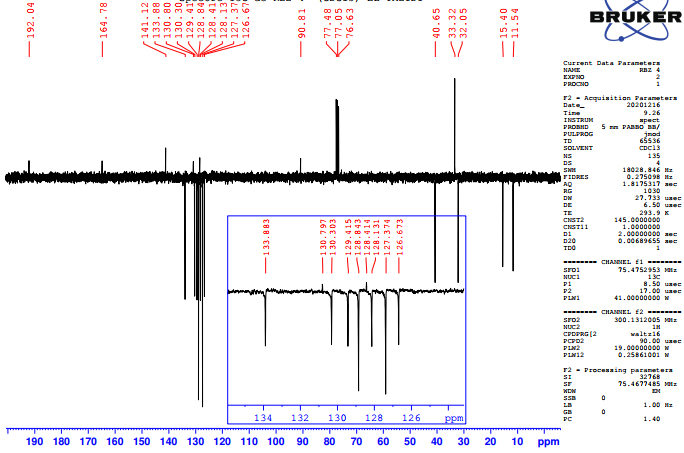


Figure 3. ^13^C NMR spectrum (75 MHz, CDCl_3_) of compound 3a


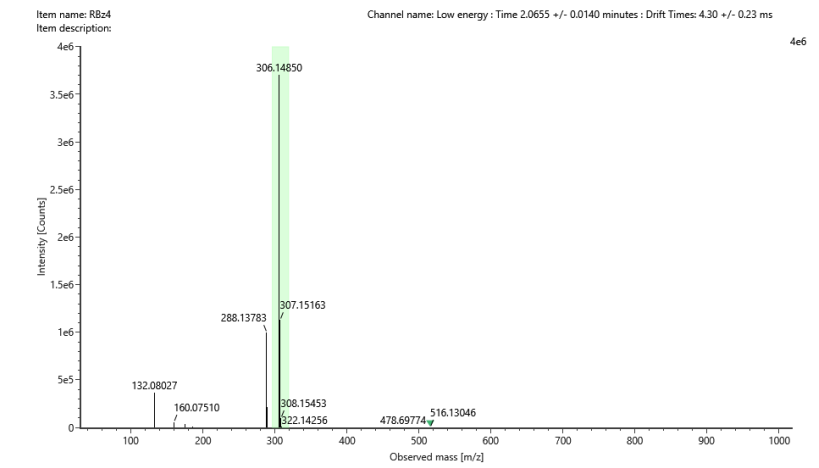


Figure 4. Mass spectrum of compound 3a

## Spectra of 3-(4-chlorophenyl)-3',4-dimethyl-3',4'-dihydro-1'H,4H-spiro[isoxazole-5,2'-naphthalen]-1'-one **3b**


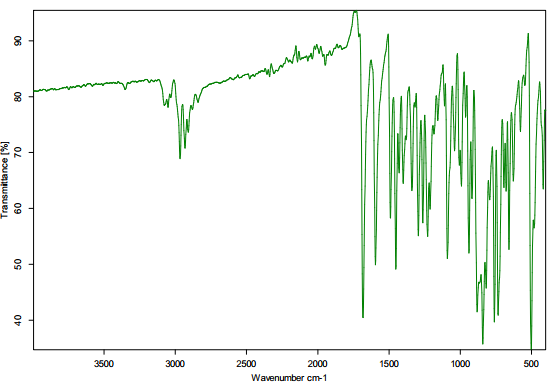


Figure 5. IR spectra of compound 3b


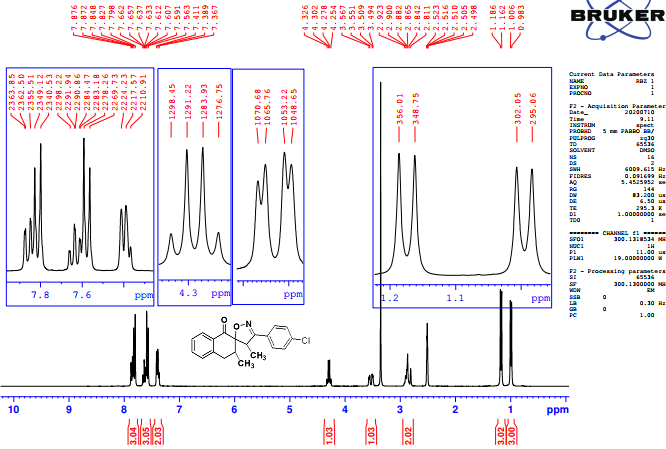


Figure 6. ^1^H NMR spectrum (300 MHz, CDCl_3_) of compound 3b


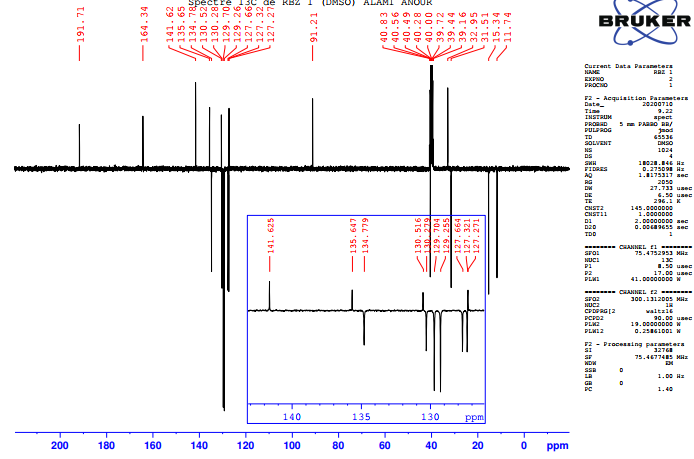


Figure 7. ^13^C NMR spectrum (75 MHz, CDCl_3_) of compound 3b


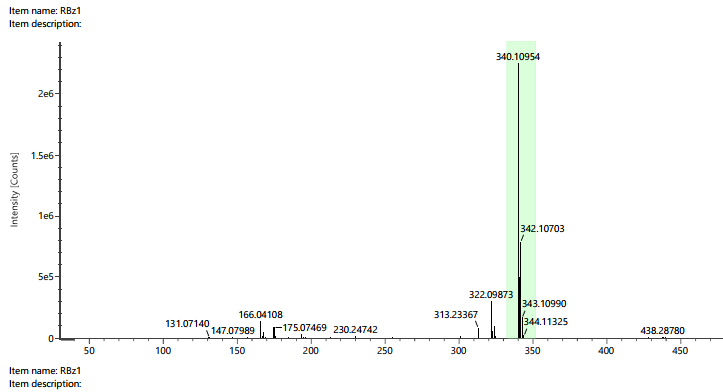


Figure 8. Mass spectrum of compound 3b

## Spectra of 3',4-dimethyl-3-(p-tolyl)-3',4'-dihydro-1'H,4H-spiro[isoxazole-5,2'-naphthalen]-1'-one **3c**


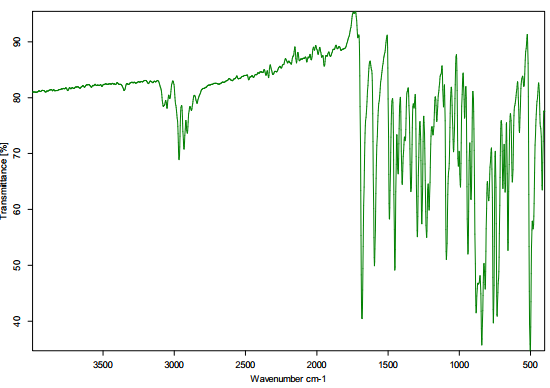


Figure 9. IR spectra of compound 3c


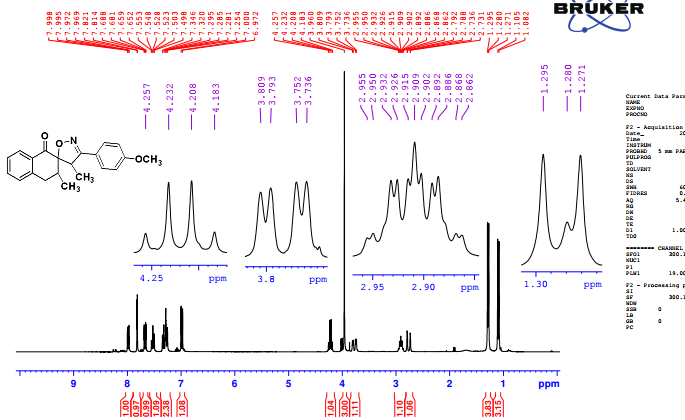


Figure 10. ^1^H NMR spectrum (300 MHz, CDCl_3_) of compound 3c


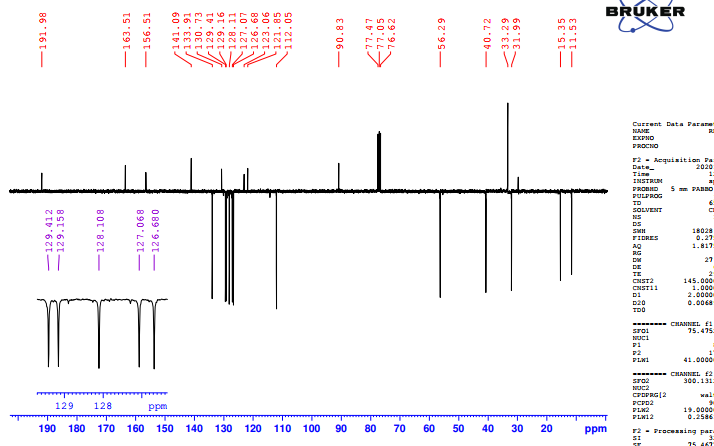


Figure 11. ^1^H NMR spectrum (75 MHz, CDCl_3_) of compound 3c


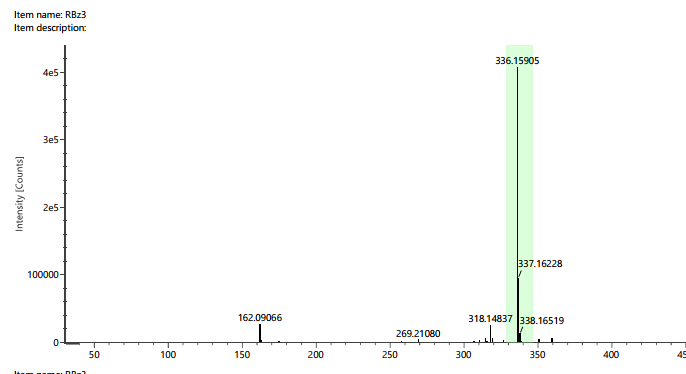


Figure 12. Mass spectrum of compound 3c

## Spectra of 3',4-dimethyl-3-(p-tolyl)-3',4'-dihydro-1'H,4H-spiro[isoxazole-5,2'-naphthalen]-1'-one **3d**


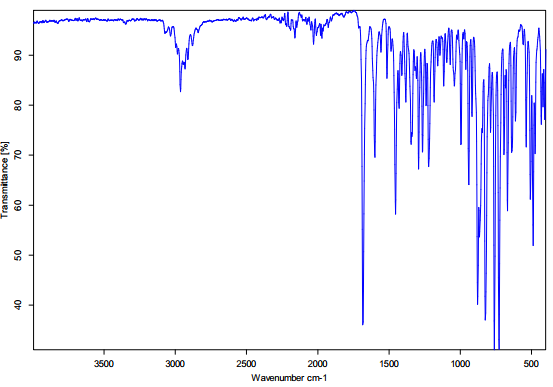


Figure 13. IR spectra of compound 3d


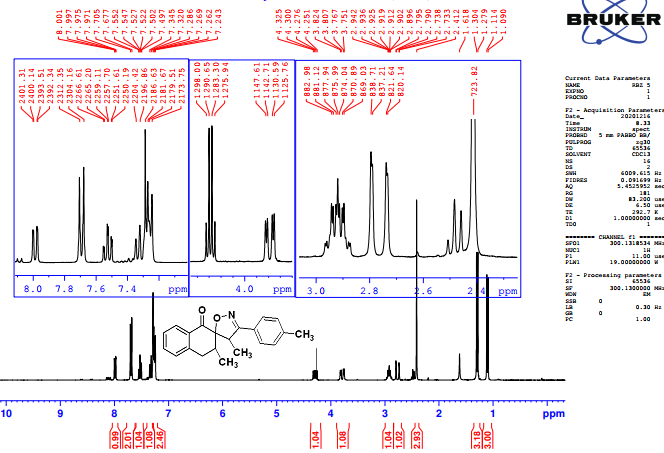


Figure 14. ^1^H NMR spectrum (300 MHz, CDCl_3_) of compound 3d


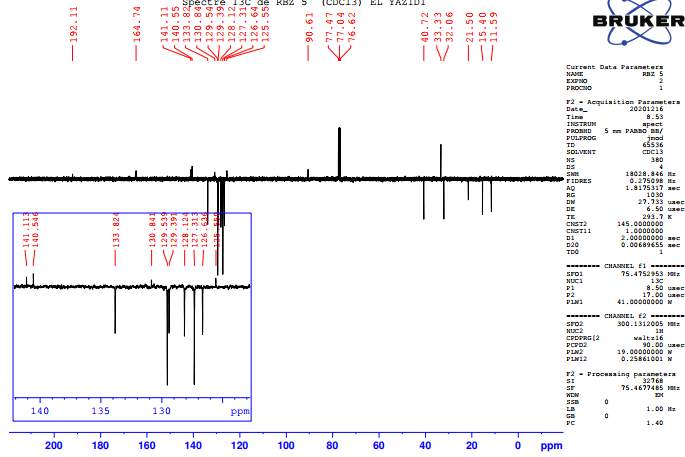


Figure 15. ^13^C NMR spectrum (75 MHz, CDCl_3_) of compound 3d


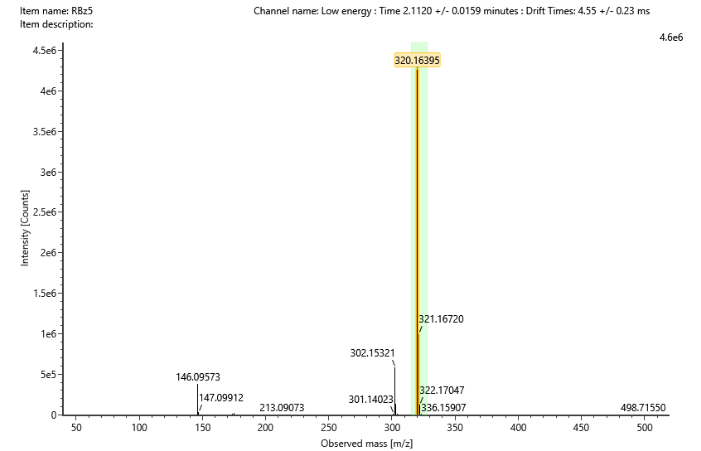


Figure 16. Mass spectrum of compound 3d.
